# Developing new tomato lines in Indigo Rose genetic background with differential anthocyanin and carotenoid production in fruits

**DOI:** 10.3389/fpls.2026.1877977

**Published:** 2026-07-13

**Authors:** Dongsheng Tian, Benny Jian Rong Sng, Ignatius Ren Kai Phang, Sing Hui Leong, Raji Mohan, Shilu Zhang, Yuejing Gui, In-Cheol Jang, Zhongchao Yin

**Affiliations:** 1 Temasek Life Sciences Laboratory, National University of Singapore, Singapore, Singapore; 2 Department of Biological Sciences, National University of Singapore, Singapore, Singapore

**Keywords:** anthocyanin, carotenoid, flavonoid, genome editing, marker-assisted selection, RNA-Seq, tomato

## Abstract

Tomato (*Solanum lycopersicum*) fruit pigmentation during ripening results primarily from carotenoid accumulation and chlorophyll degradation, with flavonoids, particularly anthocyanins in specialized genotypes, contributing to fruit coloration. To develop tomato lines with diverse pigmentation and enhanced nutritional profiles in fruits, we combined conventional breeding with CRISPR/Cas9 editing in the Indigo Rose (IR) background. Four new lines, Red Rose (RR), Pink Rose (PR), Yellow Rose (YR), and Yellow-Purple Rose (YP), were generated by knocking out *Aft* and *Myb12* genes and introducing the *PSY1/Unknown* allele. RNA-seq analysis revealed extensive tissue-specific transcriptomic remodeling, with peel and flesh diverging during ripening. Gene ontology enrichment indicated altered gene expression in flavonoid and carotenoid biosynthesis pathways. Metabolomic profiling supported these results: IR and YP peels were rich in anthocyanins, including delphinidins, petunidins and malvidins, whereas YR and YP flesh contained reduced total carotenoids but elevated α-carotene. Integrated analyses demonstrate that targeted modification of key enzyme or regulatory genes can reprogram fruit pigmentation, providing valuable genetic resources for tomato breeding.

## Introduction

Tomato (*Solanum lycopersicum*) is one of the most widely cultivated fruit vegetables worldwide, valued for both its economic significance and nutritional benefits. It serves as a rich source of vitamins, minerals, and bioactive compounds, particularly carotenoids and flavonoids, which function as antioxidants linked to a reduced risk of chronic diseases, including cardiovascular disorders and certain types of cancer (Raiola et al., 2014; Perveen et al., 2015). Among the various quality attributes of tomato, fruit coloration influences consumer preference and marketability. The development of fruit color is a genetically controlled process involving the coordinated accumulation of natural pigments, primarily carotenoids in the pericarp and flavonoids in the peel, alongside chlorophyll degradation during ripening (Kang et al., 2017; Chattopadhyay et al., 2021). Carotenoids such as lycopene and β-carotene are responsible for the red, orange, and yellow hues in the fruit flesh, while flavonoids, including naringenin chalcone and anthocyanins, contribute to the yellow and purple pigmentation of the peel. The biosynthesis of these pigments is governed by distinct yet interconnected regulatory mechanisms, in which transcription factors coordinate the expression of genes involved in their respective metabolic pathways. Both pathways are tightly regulated at the transcriptional level and are influenced by developmental and environmental signals (Zhang et al., 2021; Harbart et al., 2023). Extensive breeding and biotechnological efforts have been directed toward manipulating these pathways to generate cultivars with improved nutritional quality and diverse fruit colorations (Chattopadhyay et al., 2021; Naeem et al., 2023).

Carotenoid biosynthesis in tomato occurs in chromoplasts, which are specialized plastids derived from chloroplasts during fruit ripening (Enfissi et al., 2019; Karniel et al., 2022). The pathway is initiated by the conversion of geranylgeranyl pyrophosphate (GGPP) to phytoene, which is the first committed step catalysed by phytoene synthase (PSY). Phytoene is sequentially desaturated and isomerized by phytoene desaturase (PDS), ζ-carotene desaturase (ZDS), and carotenoid isomerase (CRTISO) to yield all-trans-lycopene, a key intermediate. Lycopene is then cyclized by lycopene β-cyclase (LCY-B) alone to produce β-carotene, or by both lycopene ϵ-cyclase (LCY-E) and LCY-B to produce β-carotene. These compounds serve as precursors for xanthophylls such as lutein, zeaxanthin, and violaxanthin, which are synthesized through the actions of β-carotene hydroxylases (CrtR-b1 and CrtR-b2) and cytochrome P450 enzymes (CYP97A3 and CYP97C). Among these steps, PSY plays a central regulatory role in determining the flux through the carotenoid biosynthetic pathway. The tomato genome harbours three *PSY* genes (*PSY1*, *PSY2*, and *PSY3*), of which *PSY1* is specifically up-regulated during fruit ripening (Giorio et al., 2008; Walter et al., 2015; Stauder et al., 2018; Karniel et al., 2022). Natural mutations in *PSY1*, as well as gene-edited knockout mutants, lead to a significant reduction in carotenoid accumulation, particularly lycopene, resulting in a yellow-fleshed fruit phenotype (Chattopadhyay et al., 2021; Yang et al., 2023). Notably, mutations in *PSY1* alleles identified in the yellow-fruited accession PI 114490 and *yellow-fruited tomato 2* (*yft2*) give rise to nonfunctional chimeric mRNA transcripts (Kang et al., 2014; Chen et al., 2019). These transcripts are generated through aberrant *trans*-splicing events during post-transcriptional processing, accompanied by suppression of the wild-type *PSY1* transcript (Kang et al., 2014; Chen et al., 2019). Interestingly, no mutations have been detected within the coding regions of these *PSY1* mutant alleles. Instead, sequence variation between mutant and wild-type alleles is limited to a single nucleotide polymorphism (SNP; A-to-G) located in the fourth intron, along with a simple sequence repeat (SSR) exhibiting variation in AT repeat number, followed by two additional SNPs in the intergenic region (Kang et al., 2014; Chen et al., 2019). Although the molecular mechanism by which these DNA polymorphisms contribute to aberrant mRNA *trans*-splicing remains unclear, the A-to-G substitution in the fourth intron of the mutant *PSY1* allele represents a useful molecular marker for the breeding of yellow-fruited tomato lines.

Flavonoid biosynthesis in tomato is derived from the phenylpropanoid pathway (Ballester et al., 2010; Teo et al., 2022). The metabolic cascade begins with the conversion of phenylalanine to *p*-coumaroyl-CoA via the sequential actions of phenylalanine ammonia-lyase (PAL), cinnamate 4-hydroxylase (C4H), and 4-coumarate:CoA ligase (4CL). Chalcone synthase (CHS) catalyses the condensation of *p*-coumaroyl-CoA and malonyl-CoA to form chalcone, which is then isomerized by chalcone isomerase (CHI) into naringenin. This intermediate is hydroxylated by flavanone 3-hydroxylase (F3H) to generate dihydrokaempferol, a precursor for both flavonols and anthocyanins. Flavonol synthase (FLS) converts dihydroflavonols into flavonols such as quercetin and kaempferol, while the anthocyanin branch involves dihydroflavonol reductase (DFR), anthocyanidin synthase (ANS), and UDP-glucose:flavonoid 3-O-glucosyltransferase (UFGT). The pathway is regulated by transcription factors of the MYB, bHLH, and WD40 families (Liu et al., 2018). In ripe tomato fruits, the overall red coloration results from the combined accumulation of red carotenoids in the flesh and yellow flavonoids, primarily naringenin chalcone, in the peel. The *Myb12* gene that encodes an R2R3-MYB transcription factor is known to positively regulate the flavonoid biosynthetic pathway, specifically promoting the accumulation of naringenin chalcone in the peel (Ballester et al., 2010). A recessive allele, *y*, derived from *Solanum chmielewskii*, disrupts this regulation, preventing naringenin chalcone accumulation in the epidermis (Ballester et al., 2010). This loss of yellow flavonoids in the peel results in a pink fruit phenotype. Genome editing approaches have successfully recapitulated this phenotype by knocking out *Myb12* (Deng et al., 2018; Zhu et al., 2018; Yang et al., 2023).

Anthocyanins, a subclass of flavonoids, are responsible for purple pigmentation observed in various fruits and vegetables (Mazza and Miniati, 2018). However, most cultivated tomato varieties contain negligible amounts of anthocyanins in their fruit peel. Over the past two decades, both conventional breeding and transgenic strategies have been employed to develop anthocyanin-enriched tomato lines (Butelli et al., 2008; Mes et al., 2008; Gonzali et al., 2009; Zhang et al., 2015; Scarano et al., 2018; Sun et al., 2020). One such cultivar, ‘Indigo Rose’ (IR), producing anthocyanin-rich purple-skinned fruits, was developed by introgression of the *Anthocyanin fruit* (*Aft*) allele from *S. chilense* and the *atroviolacium* (*atv*) allele from *S. cheesmaniae* into the cultivated tomato genome (Mes et al., 2008; Gonzali et al., 2009). *Aft* encodes an AN2-like R2R3-MYB transcription factor that activates anthocyanin biosynthesis in a light-dependent manner, while the dominant *Atv* allele in cultivated tomatoes encodes a repressor (Sun et al., 2020; Yan et al., 2020). In IR, the presence of a non-functional *atv* allele lifts this repression, and the combined effect of *Aft* and *atv* synergistically enhances anthocyanin accumulation in the peel (Cao et al., 2017; Colanero et al., 2018; 2020; Sun et al., 2020; Yan et al., 2020). Furthermore, ectopic expression of *Aft* under the control of the fruit ripening-specific *E8* promoter can drive anthocyanin accumulation in both the peel and flesh (Sun et al., 2020).

Advances in transcriptomic and metabolomic technologies have substantially enhanced our understanding of the regulatory networks and metabolic fluxes underlying pigment biosynthesis in tomato. High-throughput RNA sequencing (RNA-seq) has identified key regulatory genes and expression patterns linked to pigment accumulation and color development (Wang et al., 2019; Sun et al., 2020). Concurrently, comprehensive metabolomic analyses have facilitated the detailed profiling of carotenoid and flavonoid contents across different genotypes, thereby providing valuable insights for precision breeding aimed at improving fruit quality and pigmentation diversity (Raiola et al., 2014; Zhu et al., 2018). Our previous work demonstrated that the anthocyanin content in IR fruits was significantly higher when the plants were grown in a controlled growth chamber under a long-day photoperiod and ambient temperature than when they were grown in a greenhouse under a short-day photoperiod and elevated temperature (Teo et al., 2022). Given its distinctive purple appearance, high yield potential, and environmental adaptability, IR represents a valuable genetic background for further improvement. In this study, by combining conventional breeding and CRISPR/Cas9 genome editing, we developed new tomato lines in the IR genetic background that exhibit differential accumulation of anthocyanins and carotenoids. We further characterized their distinct pigment profiles and the molecular basis of pigment biosynthesis through transcriptomic and metabolomic analyses. Ultimately, these isogenic tomato lines serve as valuable genetic resources for the development of high-quality cultivars specifically adapted to controlled-environment agriculture.

## Materials and methods

### Plant materials, growth conditions, and fruit sampling

Seeds of *Solanum lycopersicum* cv. Indigo Rose (IR) were obtained from Plant World Seeds (https://www.plant-world-seeds.com/store/vegetable_seeds/tomato_seeds). IR18 is an introgression line in the IR genetic background carrying the *Psy1/Unknown* allele (Yuan et al., 2008; Kang et al., 2014). To develop a near-isogenic line, designated Purple-Yellow Rose (PY), IR18 was further backcrossed to IR for two additional generations, followed by self-pollination. All plants, including genome-edited lines, were cultivated in a hydroponic system under controlled environmental conditions in growth chambers. The photoperiod was set to 14 h light/10 h dark, with a photosynthetic photon flux density (PPFD) of 300 µmol m^-^² s^-^¹. Temperature and relative humidity were maintained at 22 °C and 65%, respectively. Tomato fruits were harvested at 32 (Stage 1), 40 (Stage 2), 48 (Stage 3) or 56 (Stage 4) days post-anthesis (dpa) for Brix Index determination (Stage 4), RNA sequencing (Stages 1 to 3), qRT-PCR (Stage 3) and metabolomic analyses (Stages 3 and 4). These developmental stages correspond to DS08, RS02 (Breaker), RS04 (Pink) and RS06 (Red Ripe) stages of red tomato fruit development, as defined by Skolik et al. (2019) (Skolik et al., 2019). For fruit tissue sampling, the peel corresponded to the exocarp, whereas the flesh comprised the mesocarp and inner pericarp tissues.

### Determination of total soluble solids content

Tomato fruits were harvested at 56 dpa (Stage 4) for the determination of total soluble solids (TSS) content. Fruit juice was extracted by direct squeezing, and TSS was measured using a handheld refractometer (R9500, REED Instruments). Results were expressed in degrees Brix (°Brix).

### Tomato transformation

Germinated tomato seedlings were cultivated under sterile conditions in a tissue culture facility at a controlled temperature of 25 °C, with a photoperiod of 16 h light and 8 h dark. Agrobacterium-mediated transformation was performed using cotyledon and hypocotyl explants derived from 10-day-old seedlings, following the protocol described by El-Siddig et al. (2009) with a slight modification (El-Siddig et al., 2009). The explants were initially cultured on preculture medium consisting of Murashige and Skoog (MS) basal salts supplemented with 1 mg/L 6-benzylaminopurine (6-BA) and 1 mg/L naphthaleneacetic acid (NAA) (pH 5.8) for two days. Following preculture, the explants were immersed in an *Agrobacterium* suspension, briefly blotted on sterile filter paper, and subsequently transferred to co-cultivation medium (MS medium supplemented with 1 mg/L 6-BA, 1 mg/L NAA, and 0.2 mM acetosyringone, pH 5.8) for a two-day co-cultivation period in darkness. After co-cultivation, explants were transferred to selection medium [MS medium supplemented with 2.5 mg/L 6-BA, 0.1 mg/L indole-3-acetic acid (IAA), 250 mg/L cefotaxime, and 12.5 mg/L hygromycin, pH 5.8] to induce shoot regeneration. Regenerated shoots were subsequently transferred to rooting medium (MS medium supplemented with 1 mg/L IAA, 250 mg/L cefotaxime, and 12.5 mg/L hygromycin, pH 5.8) for root development.

### Constructs for genome editing

**Figure 1:**
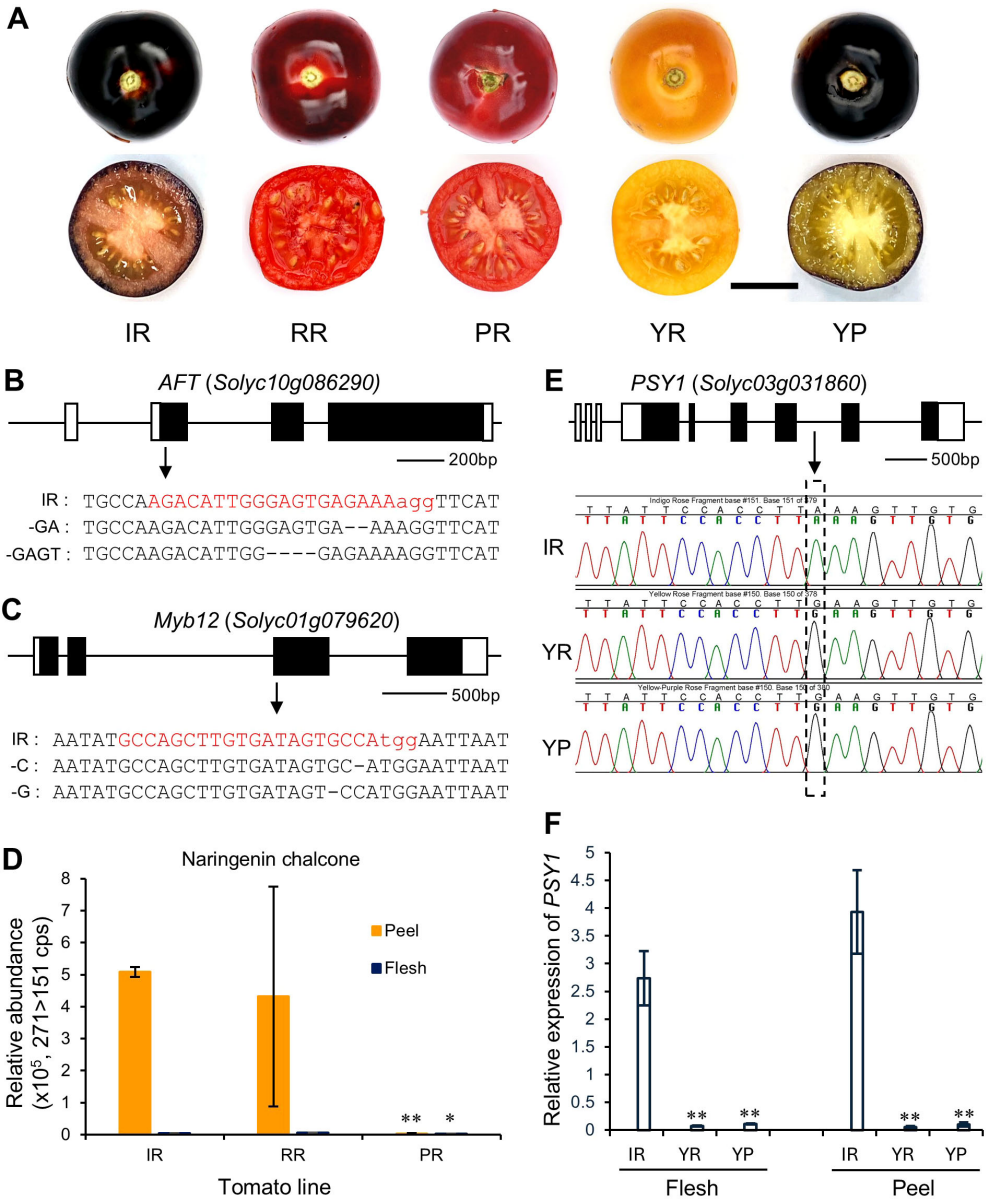
Generation of tomato lines in the Indigo Rose genetic background. **(A)** Representative images of fruit peel (upper panel) and flesh (lower panel) from Indigo Rose and its derived lines at 56 dpa. Abbreviations: IR, Indigo Rose; RR, Red Rose; PR, Pink Rose; YR, Yellow Rose; YP, Yellow-Purple Rose. Scale bar = 2 cm. **(B)** Gene structure of *Aft* and its gene-edited mutants. Schematic diagrams illustrate gene structures, with 5’ or 3’ untranslated regions (UTRs), exons, and introns represented by open boxes, filled boxes, and lines, respectively. CRISPR/Cas9 target sequences in the IR genotype are highlighted in red, and protospacer adjacent motifs (PAM) are indicated in lowercase red letters. Sequences of CRISPR/Cas9-induced mutant alleles are shown below, with deletions denoted by dashes. **(C)** Gene structure of *Myb12* and its gene-edited mutants. Diagrammatic representation and sequence annotations are as described in **(B)**. **(D)** Relative abundance of naringenin chalcone in fruit tissues, determined by UPLC-MS/MS analysis. Asterisks indicate statistically significant differences between IR and each variant (**p ≤ 0.01; *0.01 < p ≤ 0.05; Welch’s *t*-test). **(E)** Gene structure of *PSY1* and single-nucleotide polymorphisms (SNPs) in intron 4 of mutant alleles. Diagrammatic representation and sequence annotations are as described in **(B)**. The A-to-G substitutions in intron 4 distinguish the wild-type *PSY1* allele in IR from the mutant allele (*PSY1/Unknown*) in YR and YP. **(F)** Quantitative real-time PCR (qRT-PCR) analysis of wild-type *PSY1* transcript levels in peel and flesh tissues of IR, YR and YP fruits at 48 dpa. Data are presented as means ± SD of three biological replicates. Asterisks indicate statistically significant differences between IR and each variant (**p ≤ 0.01; Welch’s *t*-test).

CRISPR/Cas9-mediated genome editing was employed to generate knockout mutants of *Aft* (Solyc10g086290) and *Myb12* (Solyc01g079620). Single-guide RNAs (sgRNAs) were designed to target coding sequences within the second exon of *Aft* (5′-AGACATTGGGAGTGAGAAA-3′) and the third exon of *Myb12* (5′-GCCAGCTTGTGATAGTGCCA-3′), respectively (**Figures 1B, C**).

Guide RNA (gRNA) expression cassettes driven by the *Arabidopsis* U6–1 or U3b small nuclear RNA promoters were constructed using the pYLgRNA vector. The resulting gRNA expression cassettes were subsequently cloned into the binary vector pYLCRISPR/Cas9PUbi-H to generate the constructs pYLCas9-*Myb12* and pYLCas9-*Aft*, following the procedure described by Ma et al. (2015) (Ma et al., 2015). The constructs were introduced into *Agrobacterium tumefaciens* strain AGL1 and used for tomato transformation.

### Whole-genome sequencing of tomato lines

The genomes of all tomato lines (IR, RR, PR, YR, and YP) used or developed in this study were re-sequenced by using a whole-genome sequencing approach. Genomic DNA was extracted from young tomato leaf tissue using a standard CTAB-based extraction protocol. High-throughput sequencing was performed by BGI Genomics, generating 150-bp paired-end clean reads with an average coverage of approximately 30× genome coverage. Sequence reads were aligned to the tomato reference genome *Solanum lycopersicum* Heinz 1706 (NCBI accession GCA_000188115.3) using the BWA-MEM algorithm. The resulting alignment files were processed using SAMtools for sorting and indexing. Variant calling was performed using the Genome Analysis Toolkit (GATK) following standard best-practice workflows.

### Quantitative real-time PCR

Peel and flesh tissues were separately collected from fruits at 48 dpa (Stage 3). Total RNA was extracted from each sample using the FavorPrep Total RNA Isolation Mini Kit (FATRK001-1; Favorgen) according to the manufacturer’s instructions. First-strand cDNA was synthesized from 1 μg of total RNA using the iScript™ cDNA Synthesis Kit (Bio-Rad, USA). Quantitative real-time PCR (qRT-PCR) was performed using a Bio-Rad CFX96 Real-Time PCR System with the synthesized cDNA as the template. Relative gene expression levels were normalized to the tomato *Actin* gene (Solyc03g078400), which served as the internal reference gene. Primers for wild-type *Psy1* (5’CTGTATGGGCATCTTTGGTCTTGT3’/5’GTGGCAGTTTTTGTAGGAGGCAC3’) and *Actin* (5’GCCGCATGCCATTCTTCGT3’/5’GCTCCTGGCAGTTTCAATCTCCT3’) were obtained from a previously published study (Kang et al., 2014). Three biological replicates were analyzed for each genotype, with each replicate consisting of a single fruit harvested from an independent plant.

### RNA-Seq

Total RNAs were extracted from the peel and flesh tissues of tomato fruits harvested at 32 (Stage 1), 40 (Stage 2), and 48 (Stage 3) dpa using the FavorPrep™ Total RNA Isolation Kit (https://www.favorgen.com). The extracted RNA samples were subjected to RNA sequencing, which was performed by Novogene (https://www.novogene.com). For each sample, approximately 12 GB of raw sequencing reads were generated using the NovaSeq PE150 platform. Three biological replicates were analyzed for each condition, with each replicate consisting of a single fruit harvested from an independent plant.

### Transcriptome analysis

All gene expression data were filtered to remove genes with low average expression of fragments per kilobase transcript per million mapped reads (FPKM) < 1. To identify differentially expressed genes (DEGs) from each tomato line, their gene expression was compared to the corresponding samples of Indigo Rose (IR). DEGs were obtained by filtering for FPKM fold change > 2 or < 0.5 and *P*-value < 0.05. The DEGs were then used to generate UpSet plots using ChiPlot (https://www.chiplot.online/). Gene ontology (GO) terms for each set of DEGs were elucidated using the GO enrichment analysis tool (https://geneontology.org/) (Ashburner et al., 2000; Aleksander et al., 2023). Dot plots for comparison of GO terms were generated using R software (version 4.2.1) and ggplot2 package (https://www.rdocumentation.org/packages/ggplot2/versions/3.3.5). Raw data from RNA-sequencing (RNA-seq) were processed using MATLAB (version R2024b) to generate the principal component analysis (PCA) plot. Gene expression heat maps for carotenoid and flavonoid biosynthetic pathways were generated using Microsoft Excel.

### Quantification of total carotenoids and anthocyanins

Lyophilized tomato samples were used for the quantification of total carotenoids and relative anthocyanin levels in the peel and flesh tissues of tomato fruits at 48 (Stage 3) and 56 (Stage 4) dpa. For each genotype and developmental stage, three biological replicates were analyzed, each consisting of a single fruit harvested from an independent plant. Carotenoids were extracted at room temperature for 1 h with 100% methanol (500 µl methanol per 10 mg dry weight). Each sample was then centrifuged at 14000 rpm and 4 °C for 10 min. The resultant supernatant was transferred into a new tube, followed by further three-fold dilution before quantification. For each extract, 100 µl was measured using an Infinite M2000 microplate reader (Tecan, Switzerland) to acquire absorbance values. Absorbance values at various wavelength (666 nm, 653 nm, 470 nm) were used for the calculation of total carotenoid levels using the formula shown in (Wellburn and Lichtenthaler, 1984). For the total anthocyanin measurement, lyophilized samples were extracted overnight at 4 °C using methanol with 1% (v/v) formic acid (500 µl methanol per 10 mg dry weight). Thereafter, water:chloroform in a 1:2 ratio was added to the sample. The aqueous layer was removed for total anthocyanin and individual anthocyanin analysis. The anthocyanin extract was further diluted to 1.5-folds before 100 µl was used for measuring absorbance with an Infinite M2000 microplate reader (Tecan, Switzerland). Calculation of relative anthocyanin content was performed using the formula (A535-A650) per gram of dried weight, whereby A535 and A650 refers to the absorbance at 535 nm and 650 nm wavelengths, respectively (Deikman and Hammer, 1995).

### Ultra-high performance liquid chromatography–diode array detector–mass spectrometry analysis of individual carotenoids

The analysis of individual carotenoids was carried out using an Agilent 6495 Triple Quadrupole with 1290 Infinity II LC System equipped with a Diode Array Detector (DAD) and coupled to an Agilent Triple Quadrupole (QqQ) Mass Spectrometer (6495; Agilent). Sample separation was performed on a C30 column (2.7 µm, 100 × 2.1mm; Halo) maintained at 40 °C. Mobile phase A consisted of 100% acetonitrile and mobile phase B consisted of 100% methanol. Flow rate was maintained at 0.4 mL/min for the following elution gradient: 0–0.5 min, 30% B; 0.5–12 min, 30–90% B; 12–17 min, 90% B; 17–17.1 min, 90–30% B. After each gradient elution, 30% B was held for 3 min to re-establish the equilibrium. The column eluent was first passed through the DAD for preliminary confirmation, by monitoring absorbance at 450 nm and 471 nm (Biehler et al., 2010; Fleshman et al., 2011). Subsequently, the effluent was introduced into the mass spectrometer. Quantification was performed in ESI positive ion mode. Individual carotenoids were identified by comparing against authentic standards. Quantification of β-carotene, α-carotene, and lutein standards (Sigma, USA) was performed by monitoring the [M]+ ions: β-carotene, m/z 536.5; α-carotene, m/z 536.4; lutein, m/z 568.0. Quantification of lycopene (Sigma, USA) was determined by measuring the [M+H]+ ions with m/z 537.5.

### Ultra-high performance liquid chromatography-MS/MS analysis of naringenin chalcone

Naringenin chalcone was extracted from the plant samples in a similar manner to that used for total anthocyanin extraction. Basically, lyophilized samples were extracted overnight at 4 °C using methanol with 1% (v/v) formic acid (500 µl methanol per 10 mg dry weight). Water:chloroform in a 1:2 ratio was subsequently added to each sample. After phase separation, the aqueous phase was used for quantification of naringenin chalcone. Quantification was carried out using Agilent 6495 Triple Quadrupole with 1290 Infinity II LC System coupled to Agilent Triple Quadrupole (QqQ) Mass Spectrometer (6495; Agilent). Sample separation was performed using Luna Omega 1.6 µm Polar C18 column (1.8 μm, 100 × 2.1 mm; Phenomenex) maintained at 40 °C. Mobile phase A comprised of water with 0.1% (v/v) formic acid while mobile phase B comprised of absolute methanol with 0.1% (v/v) formic acid. Constant flow rate of 0.35 mL/min was used throughout the following elution gradient: 0–1 min, 10% B; 1–6.1 min, 10–90% B, 6.1–11.1 min, 90% B; 11.1–11.2 min, 90–10% B. After each gradient elution, 10% B was held for 4 min to re-establish the equilibrium. Identification of naringenin chalcone was performed in ESI negative ion mode by comparing against an authentic standard (PhytoLab, Germany). Naringenin chalcone was quantified using the following multiple reaction monitoring (MRM) transition: precursor ion of 271.0 m/z and product ion of 151.0 m/z.

### UPLC-orbitrap analysis of individual anthocyanins

Anthocyanin profiling was performed using the Vanquish UHPLC system (Thermo Scientific) coupled to Q Exactive Plus Hybrid Quadrupole-Orbitrap mass spectrometer (Thermo Scientific) via a heated electrospray ionization (HESI) source. 1 μL of each sample was separated with a Luna Omega 1.6 µm Polar C18 column (1.8 μm, 100 × 2.1 mm; Phenomenex) maintained at 40 °C at a constant flow rate of 0.4 mL/min. Mobile phase A consisted of 0.1% (v/v) formic acid in water, while mobile phase B consisted of 0.1% (v/v) formic acid in methanol. The gradient elution was set as the following: 0–0.5 min, 15% B; 0.5–13.0 min, 15–30% B; 13.0–23.0 min, 30–40% B; 23.0–24.0 min, 40–95% B; 24.0–28.0, 95% B; 28.0–29.0 min, 95–15% B. After each gradient elution, 15% B was held for 4 min to re-establish the equilibrium. The mass spectrometer was operated in positive ion mode, acquiring data using a data-dependent acquisition (DDA) method over a scan range of m/z 80–1200. Full mass spectrum (MS) scans were acquired in the Orbitrap analyzer at a resolution of 70000, with an Automatic Gain Control (AGC) target set to 1 × 10^6^ ions and a maximum injection time of 100 ms. For fragmentation analysis, dd-MS^2^ scans were triggered for the top five most abundant precursor ions that met an intensity threshold of 1.0 × 10^5^. These precursors were isolated using a m/z 4.0 window and fragmented using stepped Normalized Collision Energy (NCE) at 15, 30, and 45 eV. The resulting fragment ions were analyzed in the Orbitrap at a resolution of 17500, with an AGC target of 1 × 10^5^ and a maximum injection time of 80 ms. A dynamic exclusion of 10.0 seconds was applied, and isotope exclusion was enabled. Following data acquisition, the resultant raw files (.raw) were processed using MS-DIAL software (version 5.1) for feature detection, deconvolution, and retention time alignment across all samples (Tsugawa et al., 2015). Putative annotation of anthocyanins was subsequently performed by comparing the MS/MS spectra of the samples against MS/MS library and manual annotation based on previous reported study (Mes et al., 2008; Tsugawa et al., 2015; Su et al., 2016; Wang et al., 2017, 2020). The spectra of the identified anthocyanins and their respective reference spectra from the MS/MS library are shown in **Supplementary Figure 8**.

### Statistical analysis

To analyze the statistical significance between samples, homogeneity of variance was checked using Levene’s test while sample normality was determined by Shapiro-Wilk test. Samples with equal variance and normal distribution were subjected to unpaired two-tailed Student’s *t*-test. For comparisons with unequal variance or non-normal distributions, unpaired two-tailed Welch’s *t*-test was used instead.

## Results

### Generation of tomato lines with diverse fruit colors in the Indigo Rose genetic background

A combination of traditional breeding and genome editing approaches was employed to develop tomato lines with diverse fruit colors in the IR genetic background (**Figure 1A**). Two knockout mutants of the *Aft* gene (designated -GA and -GAGT) were generated via CRISPR/Cas9-mediated genome editing (**Figure 1B**; **Supplementary Figure 1**). The -GA mutant, named Red Rose (RR) (**Figure 1A**), was selected for further analysis. Using a similar approach, two knockout mutants of the *Myb12* gene (-C and -G) were developed (**Figure 1C**; **Supplementary Figure 2**), with the -C mutant selected for crossing with RR. A double mutant carrying both *aft* and *myb12* mutations, referred to as Pink Rose (PR) (**Figure 1A**), was identified in the F2 generation. Consistent with previously reported *myb12* mutants (Deng et al., 2018; Zhu et al., 2018; Yang et al., 2023), naringenin chalcone was nearly undetectable in the peel of PR, in contrast to its accumulation in RR and IR (**Figure 1D**). In parallel, Yellow-Purple Rose (YP) line (**Figures 1A, C**), a near-isogenic line (NIL) harboring the *PSY1/Unknown* allele at the *PSY1* locus within the IR genetic background, was developed through successive backcrossing and marker-assisted selection. Subsequently, a double mutant carrying both *aft* and *PSY1/Unknown* alleles was identified in the F2 population derived from a cross between RR and YP and named as Yellow Rose (YR) (**Figures 1A, C**). Whole-genome resequencing and PCR-based genotyping confirmed that both YR and YP harbour the *PSY1/Unknown* allele containing the A-to-G mutation in intron 4 (**Figure 1E**), an (AT)_19_ SSR and two additional SNPs in the 3’ intergenic region (data not shown), consistent with previous findings (Yuan et al., 2008; Kang et al., 2014). qRT-PCR analysis with *PSY1*-specific primers demonstrated that the wild-type *PSY1* mRNA transcripts were detected in both flesh and peel tissues of IR, but not in those tissues of YP or YR (**Figure 1F**), confirming the downregulation of *PSY1* in the *PSY1/Unknown*-containing lines (Kang et al., 2014; Chen et al., 2019). The genotypes of IR and the four newly developed lines are summarized in **Table 1**.

**Table 1:** Tomato lines and genotypes.

Tomato line	Gene and genotype			
	Aft	PSY1	Myb12	Atv
Indigo Rose (IR)	Aft	PSY1	Myb12	atv
Red Rose (RR)	aft	PSY1	Myb12	atv
Pink Rose (PR)	aft	PSY1	Myb12	atv
Yellow Rose (YR)	aft	PSY1/Unknown	Myb12	atv
Yellow-Purple Rose (YP)	Aft	PSY1/Unknown	Myb12	atv

Under controlled indoor growth conditions, all five tomato lines produced fruits of comparable size and shape but exhibited distinct peel and flesh pigmentation (**Figure 1A**). IR and YP possess a dominant functional *Aft* allele and a recessive non-functional *atv* allele, which together enhance anthocyanin biosynthesis and accumulation in the fruit peel (**Figure 1A**) (Mes et al., 2008; Gonzali et al., 2009; Sun et al., 2020). In contrast, knockout of *Aft* in RR, PR, and YR significantly reduced anthocyanin biosynthesis and accumulation in the peel (**Figure 1A**). Although dark-green or purple fruit skins were evident in RR, PR, and YR at 32 dpa (Stage 1) and 40 dpa (Stage 2), purple pigmentation was substantially diminished or absent in the peel at 48 dpa (Stage 3) and 56 dpa (Stage 4) (**Supplementary Figure 3**). Furthermore, knockout of *Myb12* in PR suppressed multiple steps in the flavonoid biosynthetic pathway, resulting in the development of pink-colored fruits (**Figure 1A**) (Fernandez-Moreno et al., 2016). Given that *PSY1* plays a central role in carotenoid biosynthesis, particularly lycopene accumulation in tomato flesh, the *PSY1/Unknown* allele in YR and YP significantly impaired carotenoid biosynthesis, resulting in yellow-colored flesh (**Figure 1A**) (Yuan et al., 2008; Kang et al., 2014). Notably, this mutation did not interfere with *Aft*- and *atv*-mediated anthocyanin biosynthesis in the peel, allowing YP fruits to exhibit a unique combination of purple peel and yellow flesh (**Figure 1A**). Although RR, PR, and YR did not develop purple fruits at 48 or 56 dpa, their peels exhibited dark green to purple pigmentation at 32 to 40 dpa (**Supplementary Figure 3**). In addition, these lines displayed purple stems and leaves, similar to IR and YP (**Supplementary Figure 4**), which is a characteristic trait of *atv* plants (Mes et al., 2008; Colanero et al., 2018). The total soluble solids (TSS) content in tomato fruits, primarily consisting of sugars such as glucose, fructose, and sucrose, serves as a key indicator of fruit quality and flavour. Higher TSS levels are generally associated with greater sweetness and more desirable flavour profiles. Although statistically significant differences in Brix degrees were detected between IR and YP, and between IR and PR, all five lines exhibited relatively low TSS levels and were not perceived as particularly sweet (**Supplementary Figure 5**).

### RNA-sequencing of tomato lines

To elucidate the molecular mechanisms underlying the distinct fruit colorations, we conducted RNA-seq of peel and flesh tissues from each tomato line at different stages of fruit development. The RNA-seq analysis primarily focused on fruits at 32, 40 and 48 dpa corresponding to Stages 1 to 3, when pigment biosynthesis and accumulation are most active (**Supplementary Figure 3**). Principal component analysis (PCA) of the RNA-seq data confirmed high reproducibility among biological replicates (**Supplementary Figure 6**). Notably, peel and flesh samples formed two clearly distinct clusters with minimal overlap in the PCA plot, reflecting the tissue-specific transcriptome profiles. Furthermore, data points corresponding to earlier developmental stages (Stage 1 and Stage 2) clustered closely together, whereas Stage 3 samples were more dispersed, forming separate clusters for peel and flesh (**Supplementary Figure 6**). This observation indicates that transcriptomic divergence between peel and flesh increases significantly as fruit development progresses. Stage 3 corresponds to the ‘pink stage’ in red-fruited tomatoes, a critical transition during which ripening initiates and final fruit coloration begins to emerge (Skolik et al., 2019). Thus, the pronounced transcriptomic changes observed at this stage likely reflect the tissue-specific activation of pigment biosynthesis pathways (**Supplementary Figure 6**).

**Figure 2:**
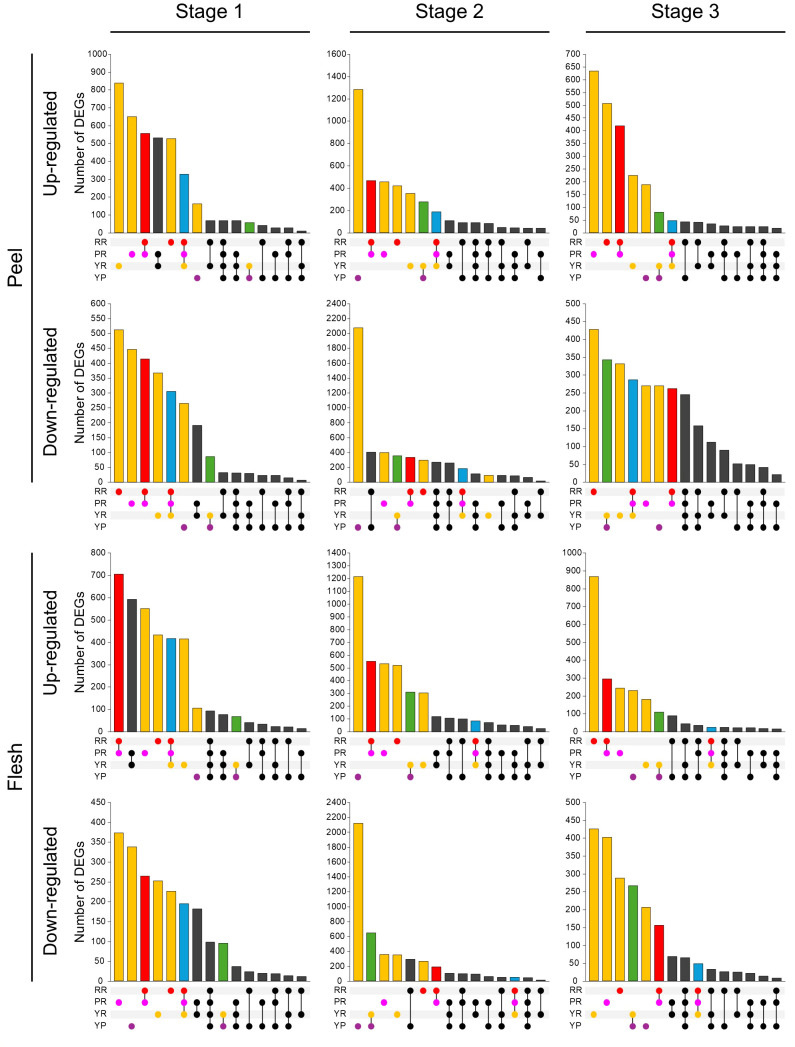
UpSet plot showing the number of DEGs specific to each tomato line or shared among multiple lines. DEGs were identified by comparison with IR. Yellow bars indicate DEGs specific to a single tomato line. Red bars represent DEGs shared between RR and PR. Blue bars represent DEGs shared among RR, PR, and YR. Green bars represent DEGs shared between YR and YP. IR, Indigo Rose; RR, Red Rose; PR, Pink Rose; YR, Yellow Rose; YP, Yellow-Purple Rose.

To further investigate these transcriptomic differences, we identified differentially expressed genes (DEGs) for each of the tomato lines. Genes with extremely low expression (FPKM < 1) were excluded. DEGs were identified by pairwise comparisons within the same tissue type and developmental stage, using the IR line as a reference. Genes were considered differentially expressed if they exhibited a fold change > 2 or < 0.5 and a *P*-value < 0.05. The resulting DEGs across all tomato lines were visualized using UpSet plots to illustrate the number of genes that were line-specific or shared among multiple lines (**Figure 2**). The number of DEGs commonly shared among all four lines (RR, PR, YR, and YP) was consistently low across all tissues and stages, indicating substantial transcriptomic divergence among the lines (**Figure 2**). This variation is likely reflected in their metabolomic profiles (see below). In fact, line-specific DEGs represented the majority of DEGs across most tissue–stage combinations (yellow bars, **Figure 2**). Despite overall divergence, a substantial proportion of the total DEGs was shared between several tomato lines (red, blue, and green bars, **Figure 2**). For instance, the DEGs common to RR and PR were likely associated with their *aft* mutation and a functional *PSY1* allele, which possibly resulted in their phenotype of red flesh combined with yellow or colorless peel that lacked the purple pigmentation characteristic of anthocyanin accumulation (red bars, **Figure 2**). Similarly, DEGs shared among RR, PR, and YR may have been derived from the *aft* mutation (blue bars, **Figure 2**). In contrast, the DEGs shared between YR and YP, both of which possess the *PSY1/Unknown* allele and produce yellow flesh, likely reflect perturbations in carotenoid biosynthesis (green bars, **Figure 2**).

Further analysis of the UpSet plots revealed that the YP line exhibited pronounced transcriptomic changes specifically at Stage 2. A large number of up- and down-regulated DEGs were identified in both peel (1,283 up-regulated; 2,071 down-regulated) and flesh (1,213 up-regulated; 2,119 down-regulated) tissues (**Figure 2**). This finding was corroborated by the PCA results, in which Stage 2 samples from YP (YP-P2 and YP-F2) clustered distinctly from other Stage 2 samples (**Supplementary Figure 6**). These observations suggest that the combination of *Aft* with the *PSY1/Unknown* allele in YP has a specific and strong regulatory effect on fruit development at this stage. Additionally, DEGs shared between RR and PR constituted the largest category of up-regulated genes in Stage 1 flesh samples (red bar, **Figure 2**), a trend not observed in other tissues or stages. This finding implies that the up-regulated genes in Stage 1 may play important roles in red flesh development in RR and PR. Moreover, comparable numbers of down-regulated DEGs were observed in Stage 3 peel samples among the RR-PR, RR-PR-YR, and YR-YP groups (red, blue, and green bars, **Figure 2**), suggesting that both *aft* and *PSY1/Unknown* alleles may have similar transcriptomic effects in peel tissues at this stage.

### Gene ontology terms reveal unique physiology in each tomato line

**Figure 3:**
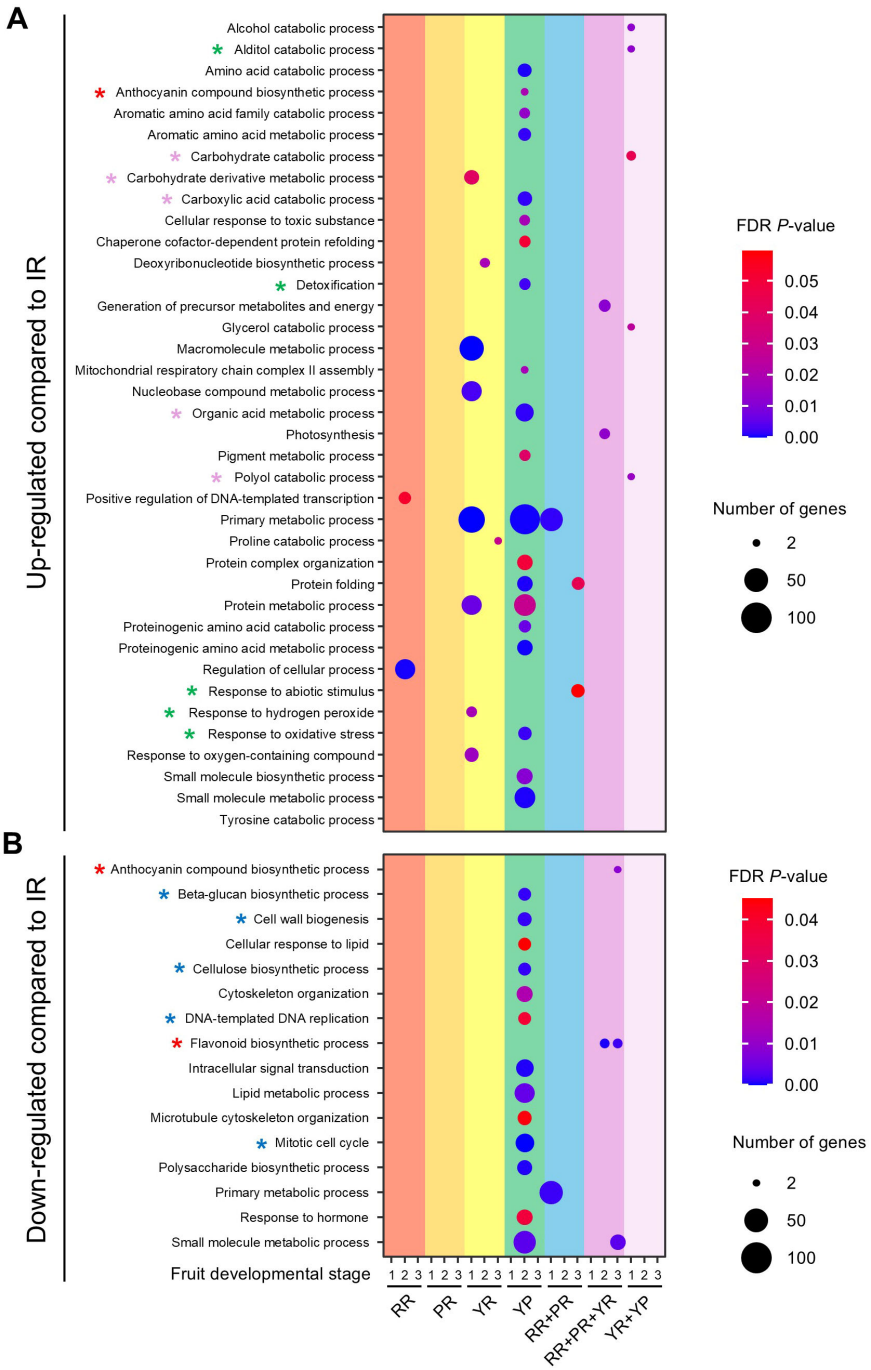
Dot plot of enriched GO terms in tomato peel samples. Significantly up-regulated **(A)** or down-regulated **(B)** GO terms in the peel of each tomato line, or shared among multiple lines, are shown relative to IR. Asterisks indicate GO terms associated with specific biological pathways: red, flavonoid biosynthesis; blue, plant cell division and cell wall organization; pink, carbohydrate and organic acid metabolism; green, stress responses. IR, Indigo Rose; RR, Red Rose; PR, Pink Rose; YR, Yellow Rose; YP, Yellow-Purple Rose; FDR, false discovery rate.

**Figure 4:**
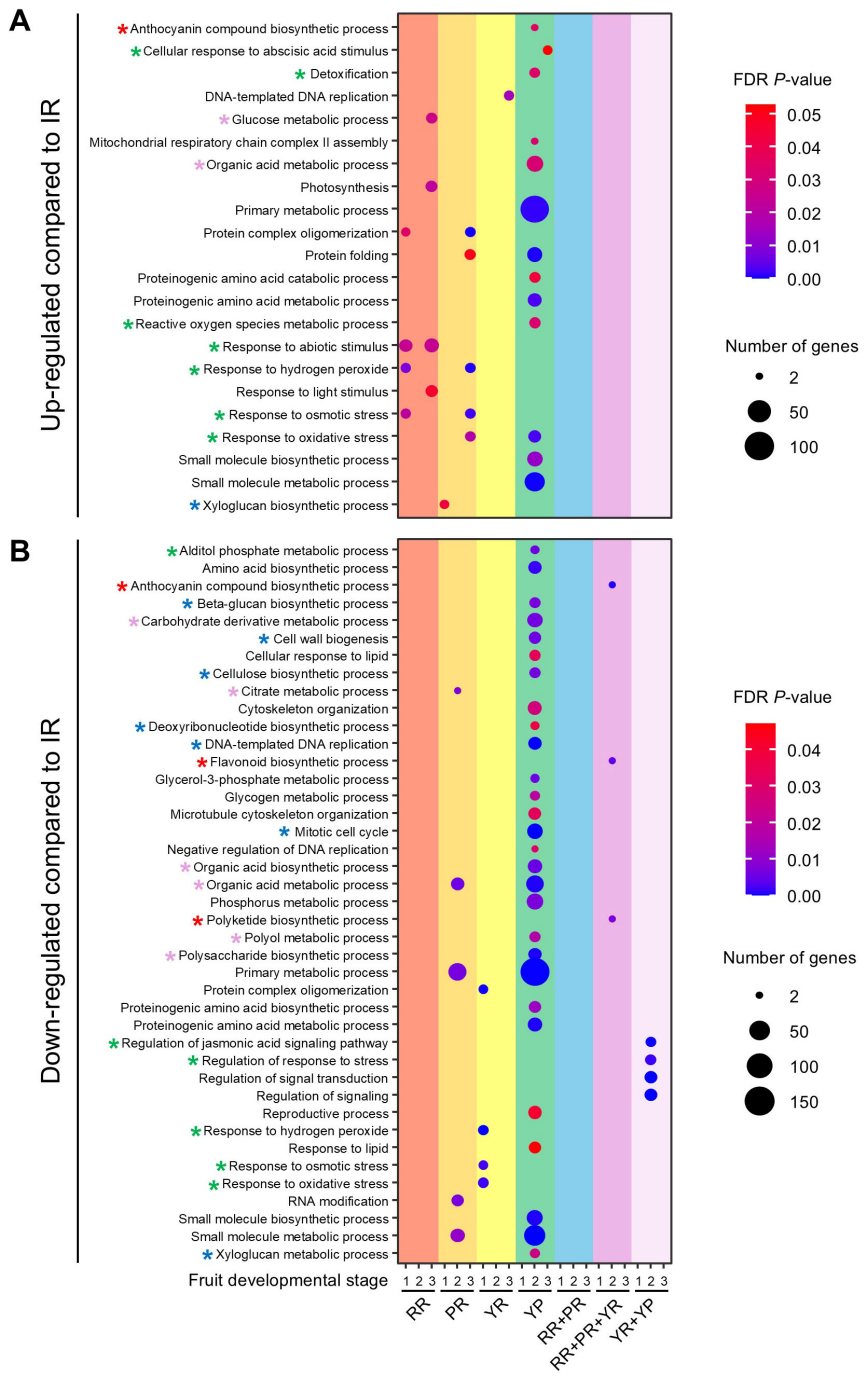
Dot plot of enriched GO terms in tomato flesh samples. Significantly up-regulated **(A)** or down-regulated **(B)** GO terms in the flesh of each tomato line, or shared among multiple lines, are shown relative to IR. Asterisks indicate GO terms associated with specific biological pathways: red, flavonoid biosynthesis; blue, plant cell division and cell wall organization; pink, carbohydrate and organic acid metabolism; green, stress responses. IR, Indigo Rose; RR, Red Rose; PR, Pink Rose; YR, Yellow Rose; YP, Yellow-Purple Rose; FDR, false discovery rate.

We further performed GO term analyses to investigate the biological processes associated with these DEGs (**Figures 3**, **4**). In tomato peels, many of the elucidated GO terms were found in YP at Stage 2 (**Figure 3**), which correlates with its large number of DEGs (**Figure 2**). Several GO terms were closely related in biological functions, including anthocyanin and flavonoid biosynthesis (red stars), carbohydrate and acid metabolism (pink stars), stress response (green stars), and cell division (blue stars) (**Figure 3**).

**Figure 5:**
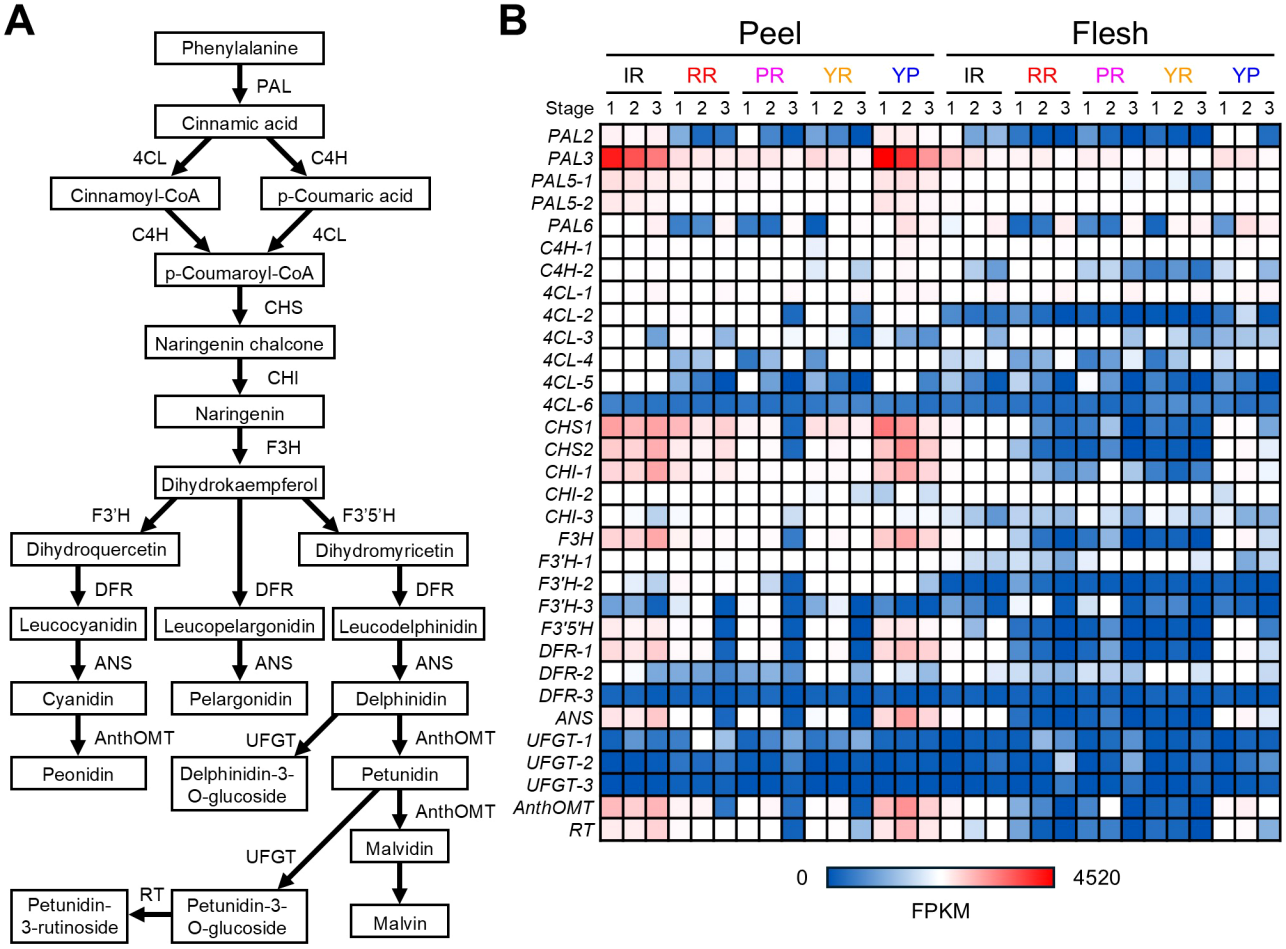
Expression of flavonoid biosynthesis genes in tomato fruits. **(A)** Schematic representation of the flavonoid biosynthesis pathway in tomato. Abbreviations: ANS, anthocyanin synthase; AnthOMT, anthocyanin-targeting O-methyltransferase; CHI, chalcone isomerase; CHS, chalcone synthase; C4H, cinnamate 4-hydroxylase; DFR, dihydroflavonol 4-reductase; F3H, flavanone 3-hydroxylase; F3’5’H, flavonoid 3’,5’-hydroxylase; F3’H, flavonoid 3’-hydroxylase; PAL, Phenylalanine ammonia-lyase; RT, rhamnosyltransferase; UFGT, UDP-glucose:flavonoid-3-O-glucosyltransferase; 4CL, 4-coumarate:CoA ligase. **(B)** Heatmap depicting the expression levels of genes involved in the pathway shown in **(A)**. IR, Indigo Rose; RR, Red Rose; PR, Pink Rose; YR, Yellow Rose; YP, Yellow-Purple Rose; FPKM, fragments per kilobase of transcript per million mapped reads.

Anthocyanin and flavonoid biosynthesis were up-regulated in YP peels at Stage 2, but down-regulated in RR, PR, and YR at Stages 2 and 3 (**Figure 3**). This finding is consistent with the peel colors observed in RR, PR, and YR, which lacked the distinctive purple coloration that is associated with anthocyanin content (**Supplementary Figure 3**). GO terms related to carbohydrate and acid metabolism were generally enriched in YR and YP peels at Stages 1 and 2, with most GO terms involving catabolic processes (**Figure 5**). Interestingly, oxidative stress-related GO terms such as “response to oxidative stress” and “detoxification” were also enriched in YP (**Figure 3**). This suggests that YP may contain higher levels of antioxidants than the other tomato varieties.

In flesh samples, YP at Stage 2 likewise contained the largest number of enriched GO terms (**Figure 4**), which is consistent with the number of GO terms in peel samples (**Figure 3**). Biological functions that were elucidated from peel samples were also identified in flesh samples, but in a different context. Similar to peels, anthocyanin and flavonoid biosynthesis were up-regulated in YP flesh while down-regulated in RR, PR, and YR flesh (**Figure 4**). However, these GO terms were down-regulated only in Stage 2 flesh samples, instead of Stages 2 and 3 as seen in the peels (**Figures 3**, **4**). GO terms related to oxidative stress like “response to hydrogen peroxide” and “response to oxidative stress” were found to be up-regulated in the flesh of RR, PR, and YP, while down-regulated in YR (**Figure 4**), indicating varying amounts of antioxidants in the flesh of these lines. Interestingly, the down-regulated DEGs shared by YR and YP were associated with jasmonic acid signalling (**Figure 4**), potentially because of their shared *PSY1/Unknown* allele. Although carotenoid content is not known to influence jasmonic acid signalling, methyl jasmonate was reported to induce carotenoid biosynthesis (Luo et al., 2020). As such, it is possible that carotenoid content may affect jasmonic acid pathways in plants as part of a feedback response.

### Differential expression of flavonoid and anthocyanin biosynthetic genes in fruits of tomato lines

Given the marked differences in fruit and peel coloration among the tomato lines (**Figure 1A**), and the altered transcriptome of the anthocyanin biosynthetic pathway in certain lines (**Figures 3**, **4**), we further examined the expression profiles of genes involved in flavonoid and anthocyanin biosynthesis. In the phenylpropanoid pathway, phenylalanine is converted through a series of enzymatic reactions into *p*-coumaroyl-CoA, which serves as the initial substrate for the flavonoid biosynthesis pathway (**Figure 5A**). This intermediate is subsequently transformed into flavones, flavonols, and anthocyanins through a cascade of downstream enzymatic steps (**Figure 5A**) (Fernandez-Moreno et al., 2016).

To visualize the expression patterns of individual genes across different lines, we generated a heatmap of their FPKM values (**Figure 5B**). Overall, IR and YP lines exhibited higher expression of flavonoid biosynthetic genes compared to other lines, particularly in peel tissues (**Figure 5B**). Notably, elevated expression was observed for genes *PHENYLALANINE AMMONIA-LYASE 3* (*PAL3*), *CHALCONE SYNTHASE 1/2* (*CHS1/2*), *CHALCONE ISOMERASE* (*CHI-1*), *FLAVANONE 3-HYDROXYLASE* (*F3H*), *FLAVONOID 3′,5′-HYDROXYLASE* (*F3′5′H*), *DIHYDROFLAVONOL 4-REDUCTASE* (*DFR-1*), *ANTHOCYANIN SYNTHASE* (*ANS*), anthocyanin-targeting *O-METHYLTRANSFERASE* (*AnthOMT*), and *RHAMNOSYLTRANSFERASE* (*RT*) (**Figure 5B**). These genes contribute to the biosynthesis of delphinidin, which is further modified into petunidin and malvidin (**Figure 5A**), the two predominant anthocyanins found in purple-colored tomatoes (Jones et al., 2003; Colanero et al., 2020). Accordingly, the high expression of these genes in IR and YP peels at developmental Stages 1 to 3 likely promoted anthocyanin accumulation, leading to the purple pigmentation observed in the two lines (**Figure 1A**; **Supplementary Figure 3**).

Interestingly, the PR line uniquely exhibited low expression of *CHS1*, *CHS2*, *F3H*, *FLAVONOID 3′-HYDROXYLASE* (*F3′H-2*), and *RT* at Stage 3 (**Figure 5B**). This observation aligns with prior studies indicating that *Myb12* regulates several flavonoid biosynthetic genes, including *CHS*, *CHI*, and *F3H* (Adato et al., 2009; Ballester et al., 2010; Fernandez-Moreno et al., 2016). Our results suggest that *Myb12* exerts its regulatory function primarily at Stage 3 to enhance anthocyanin biosynthesis, which is consistent with the pigmentation changes observed in IR and YP peels (**Supplementary Figure 3**). Furthermore, the presence of the *aft* mutation in RR, PR, and YR appears to down-regulate a set of key pathway genes in both peel and flesh tissues, including *PAL2/6*, *4-COUMARATE: COA LIGASE* (*4CL-4/5*), *CHS1/2*, *CHI-1*, *F3H*, *F3′5′H*, *DFR-1*, *ANS*, *AnthOMT*, and *RT* (**Figure 5B**). These findings align with the weak or absent purple pigmentation observed in RR, PR, and YR fruits at 56 dpa (**Supplementary Figure 3**) and further support the notion that *Aft* serves as a master regulator of flavonoid and anthocyanin biosynthesis.

To explore additional branches of flavonoid metabolism, we also analyzed the expression of genes involved in peripheral pathways (**Supplementary Figure 7A**). Notably, *P-COUMARATE 3′-HYDROXYLASE* (*C3H*) was suppressed in all flesh samples but showed reduced expression only in the peels of RR, PR, and YR (**Supplementary Figure 7B**), suggesting that caffeoyl-CoA biosynthesis is restricted to the peels of IR and YP. Moreover, *3-O-GLUCOSYLTRANSFERASE* (*3GT-1*) was repressed in the flesh of RR, PR, and YR and down-regulated in Stage 3 peel samples of PR and YR (**Supplementary Figure 7B**). In contrast, other *3GT* homologs (*3GT-2/3/4*) were expressed at relatively high and consistent levels across all varieties (**Supplementary Figure 7B**). These findings suggest possible functional redundancy among *3GT* family members, which may sustain rutin production regardless of genotype.

### Differential expression of carotenoid biosynthetic genes in fruits of tomato lines

**Figure 6:**
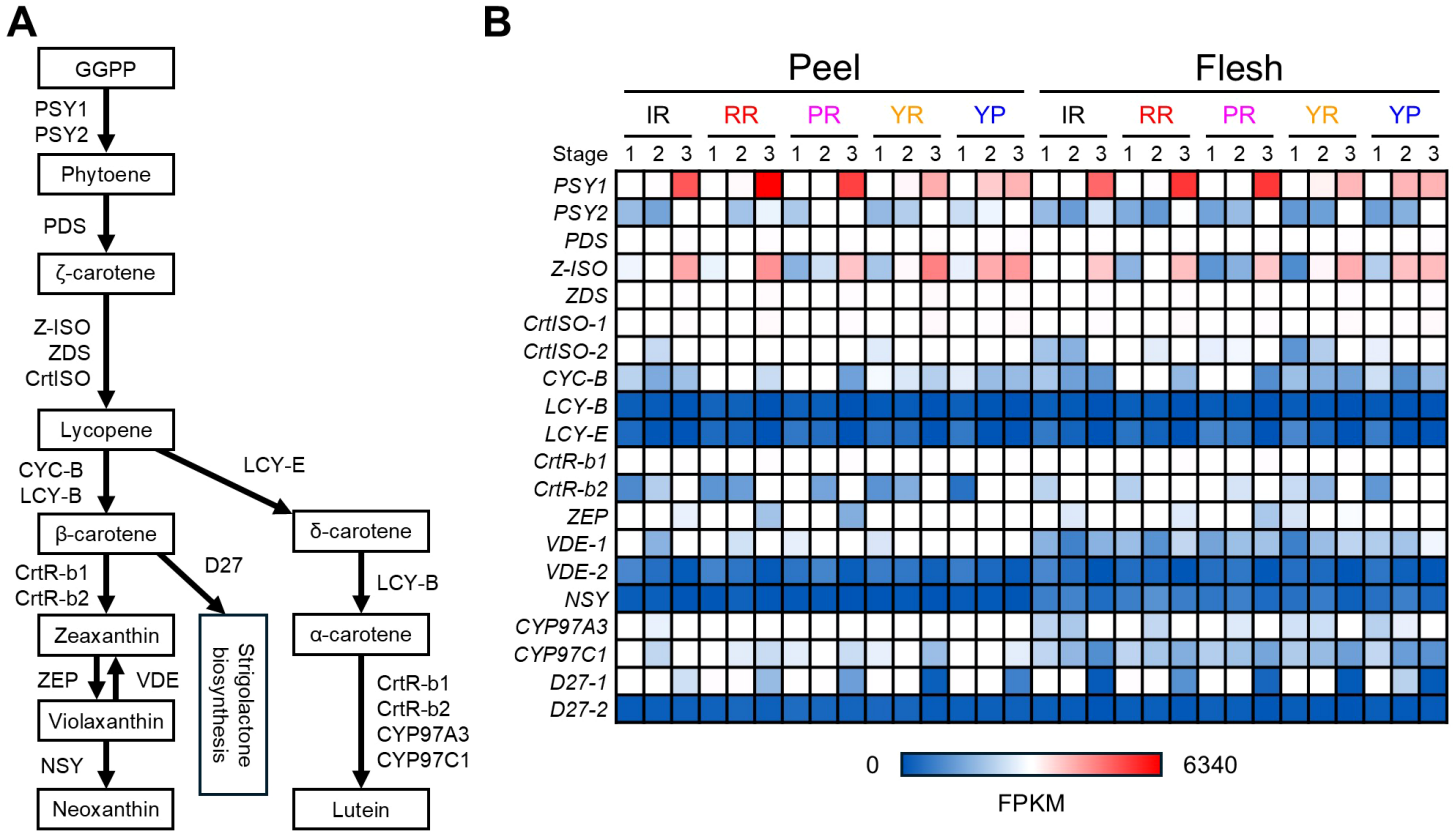
Expression of carotenoid biosynthesis genes in tomato fruits. **(A)** Schematic representation of the carotenoid biosynthesis pathway in tomato. Abbreviations: CrtISO, carotenoid isomerase; CrtR-b1, β-carotene hydroxylase 1; CrtR-b2, β-carotene hydroxylase 2; CYC-B, lycopene β-cyclase; CYP97A3, cytochrome P450 monooxygenase 97A3; CYP97C1, cytochrome P450 monooxygenase 97C1; D27, DWARF27; GGPP, geranylgeranyl di-phosphate; LCY-B, lycopene β-cyclase; LCY-E, lycopene ϵ-cyclase; NSY, neoxanthin synthase; PDS, phytoene desaturase; PSY1, phytoene synthase 1; PSY1, phytoene synthase 2; VDE, violaxanthin de-epoxidase; ZDS, ζ-carotene desaturase; ZEP, zeaxanthin epoxidase; Z-ISO, ζ-carotene isomerase. **(B)** Heatmap illustrating the expression levels of the genes depicted in **(A)**. IR, Indigo Rose; RR, Red Rose; PR, Pink Rose; YR, Yellow Rose; YP, Yellow-Purple Rose; FPKM, fragments per kilobase of transcript per million mapped reads.

In addition to anthocyanins, carotenoids such as lycopene accumulate in tomato fruits during ripening (Jones et al., 2003). To examine carotenoid biosynthesis in each tomato line, we analyzed the expression profiles of carotenoid pathway genes in peel and flesh samples across developmental Stages 1 to 3 (**Figures 6A, B**). A strong induction of *PSY1* expression was observed in both peel and flesh tissues of most genotypes at Stage 3 (**Figure 6B**), which corresponds to the ‘pink stage’ of fruit ripening in red-fruited tomatoes and marks the transition to final fruit coloration (**Supplementary Figure 3**) (Skolik et al., 2019). However, the *PSY1* transcripts, including both the wild-type *PSY1* mRNA transcripts and the nonfunctional chimeric mRNA transcripts (Kang et al., 2014; Chen et al., 2019), was only moderately induced in YR at Stage 3 and in YP at both Stages 2 and 3 (**Figure 6B**). These observations are consistent with the presence of the *PSY1/Unknown* allele in YR and YP, which leads to a knockdown of wild-type *PSY1* mRNA transcripts. Previous studies have shown that a complete knockout of *PSY1* abolishes phytoene and downstream carotenoid biosynthesis, although yellow pigmentation can still be observed due to the accumulation of naringenin chalcone (Karniel et al., 2022). In the case of YR and YP, the partial suppression of wild-type *PSY1* mRNA transcripts due to the *trans*-splicing during the expression of the *PSY1/Unknown* allele likely reduces, but does not eliminate, carotenoid production (also see below).

The expression of most downstream genes in the carotenoid biosynthetic pathway was relatively consistent across the tomato lines (**Figure 6B**). However, two cytochrome P450 genes, *CYP97A3* and *CYP97C1*, exhibited higher expression levels in peel than in flesh, suggesting that the conversion of α-carotene to lutein is more active in the peel. Additionally, the chromoplast-specific *LYCOPENE β-CYCLASE* (*CYC-B*), which catalyses the conversion of lycopene to β-carotene (**Figure 6A**), showed markedly higher expression in RR and PR at Stages 1 and 2 compared to the other tomato lines (**Figure 6B**). This elevated expression implies a higher accumulation of β-carotene in the fruits of RR and PR lines.

### Comparative analysis of total anthocyanin and carotenoid content in fruits of tomato lines

**Figure 7:**
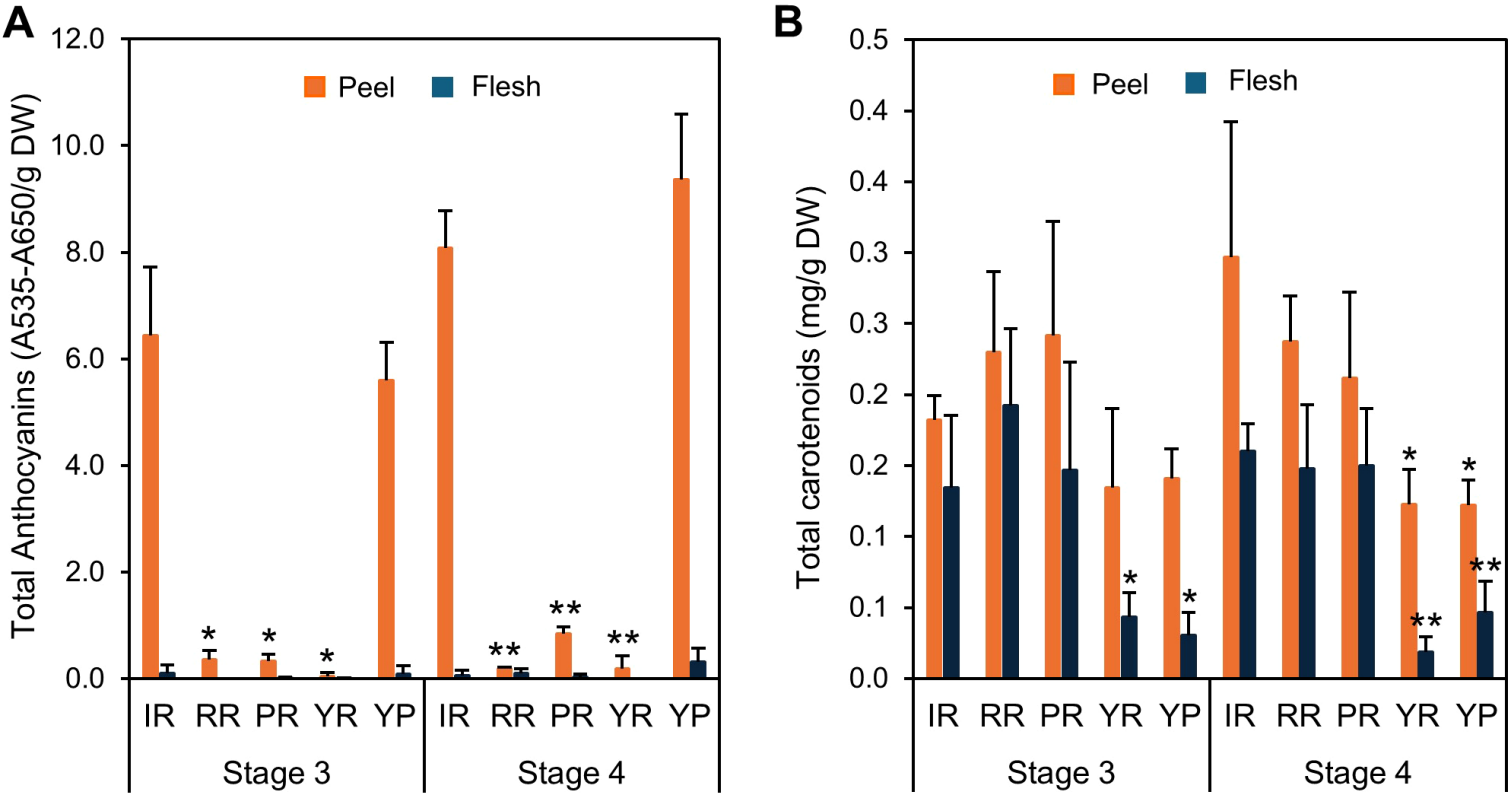
Quantification of total pigment compounds in tomato lines. Quantitative analysis of total anthocyanins **(A)** and total carotenoids **(B)** across the different tomato lines. Asterisks indicate statistically significant differences between IR and individual variant (**, p ≤ 0.01; *, 0.01 < p ≤ 0.05; Student’s *t*-test). The relative anthocyanin content is represented as (A535-A650)/g DW, whereby A535 and A650 refers to the absorbance at 535 nm and 650 nm wavelengths, respectively. IR, Indigo Rose; RR, Red Rose; PR, Pink Rose; YR, Yellow Rose; YP, Yellow-Purple Rose.

To assess whether the transcriptome data correlates with pigment accumulation in tomato fruits, we quantified total anthocyanins and carotenoids from peel and flesh tissues of each tomato line (**Figure 7**). Total anthocyanin content was highest in the peels of IR and YP, reaching approximately 6.0 (A535–A650)/g dry weight (DW) at Stage 3, and further increased to around 8.5 (A535–A650)/g DW at Stage 4 (**Figure 7A**). In contrast, anthocyanin levels in the peels of RR, PR and YR were much lower, ranging from 0.05 to 0.3 (A535–A650)/g DW at Stage 3 and 0.2 to 0.9 (A535–A650)/g DW at Stage 4, respectively (**Figure 7A**). Furthermore, minimal anthocyanin accumulation was observed in the flesh across all tomato lines, with concentrations consistently below 0.4 (A535–A650)/g DW (**Figure 7A**). These results are consistent with transcriptomic data, which showed strong expression of anthocyanin biosynthetic genes in IR and YP peels, moderate expression in RR, PR and YR peels, and low expression in all flesh samples (**Figure 5B**).

Total carotenoid content was consistently higher in the peel than in the flesh across all tomato lines (**Figure 7B**). This pattern corresponds with the elevated expression of several carotenoid biosynthetic genes, including *PSY2*, *ZETA-CAROTENE ISOMERASE* (*Z-ISO*), *CAROTENE CIS-TRANS ISOMERASE* (*CrtISO-2*), *CYC-B*, *VIOLOXANTHIN DEEPOXIDASE* (*VDE-1*), *CYP97A3*, and *CYP97C1*, providing a molecular basis for the differential accumulation observed (**Figure 6B**). Among the genotypes, IR, RR, and PR exhibited higher carotenoid accumulation in both peel and flesh tissues than YR and YP (**Figure 7B**). In IR peel, total carotenoid content increased from 0.18 mg/g DW at Stage 3 to 0.30 mg/g DW at Stage 4, whereas RR and PR peels maintained relatively high levels (~0.25 mg/g DW) at both stages (**Figure 7B**). By contrast, carotenoid content in the flesh of the three lines remained stable across stages, averaging approximately 0.20 mg/g DW (**Figure 7B**). YR and YP exhibited consistently low carotenoid levels in both peel and flesh, with flesh tissues accumulating less than 0.05 mg/g DW at both stages (**Figure 7B**). These metabolite profiles are consistent with the presence of the *PSY1/Unknown* allele in YR and YP and the concomitant reduced expression of the wild-type *PSY1* mRNA transcripts in these lines (**Figure 1F**; **Figure 6B**). As *PSY1* encodes the enzyme catalyzing the first committed step in carotenoid biosynthesis, the *PSY1/Unknown* mutant allele is responsible for the low total carotenoid accumulation observed in YR and YP (Kang et al., 2014).

### Quantification of specific anthocyanins and carotenoids in fruits of tomato lines

**Figure 8:**
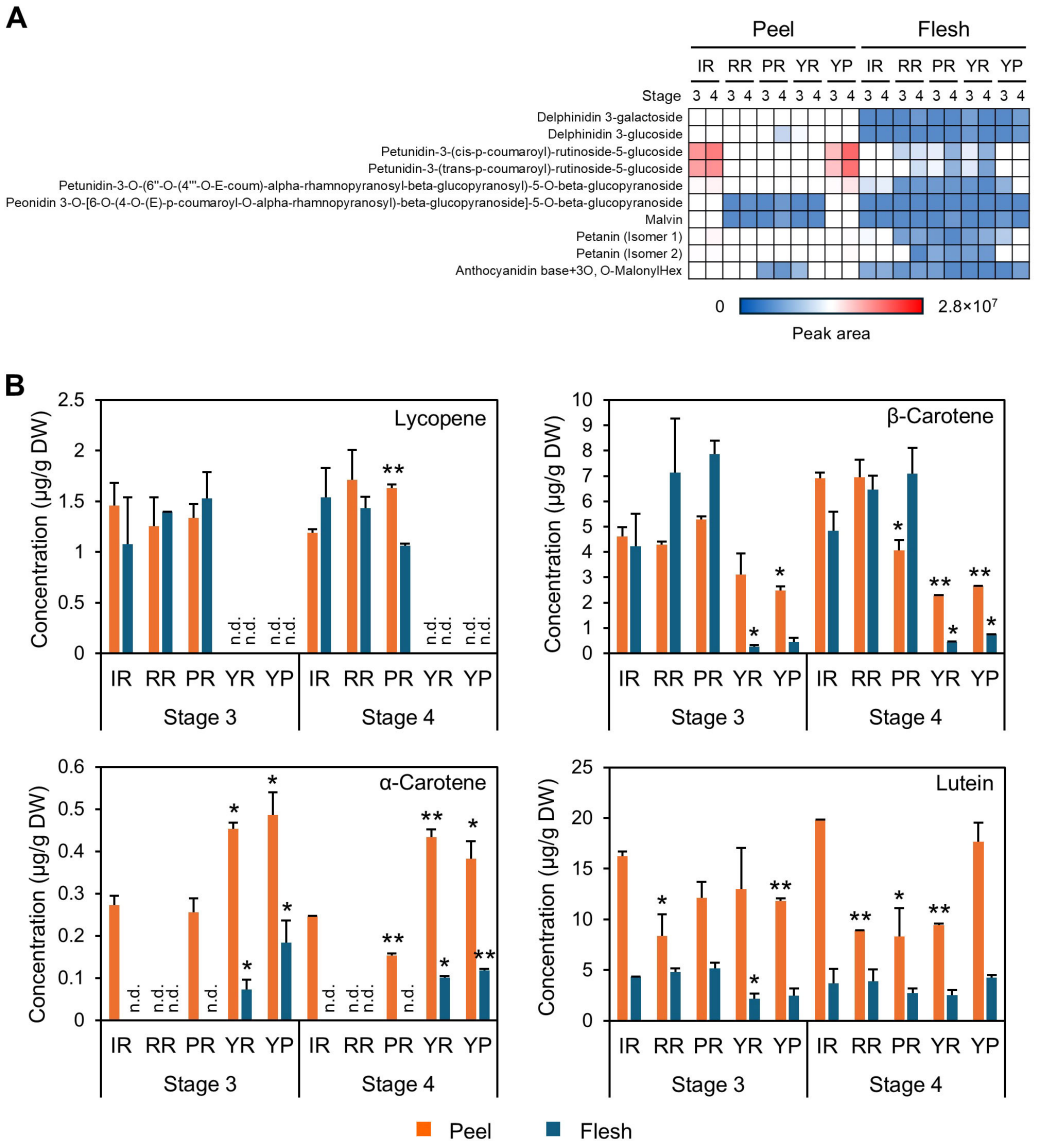
Quantification of specific pigment compounds in tomato lines. **(A)** Heatmap representing untargeted analysis of anthocyanins performed by LC-MS/MS. Warmer colors correspond to larger peak areas, indicating higher anthocyanin abundance. IR, Indigo Rose; RR, Red Rose; PR, Pink Rose; YR, Yellow Rose; YP, Yellow-Purple Rose. **(B)** Targeted quantification of specific carotenoids by LC-MS/MS. Asterisks indicate statistically significant differences between IR and individual variant (**p ≤ 0.01; *0.01 < p ≤ 0.05; Student’s *t*-test). n.d., not detected.

To further characterize pigment composition in tomato fruits of various genotypes, we employed liquid chromatography–tandem mass spectrometry (LC-MS/MS) to identify and quantify specific anthocyanins and carotenoids. Untargeted LC-MS/MS analysis revealed several anthocyanin classes, including delphinidins, petunidins, peonidins, and malvidins (**Figure 8A**; **Supplementary Figure 8**). These compounds were generally more abundant in the peel tissues than in the flesh, as illustrated by the heatmap visualization of chromatographic peak areas (**Figure 8A**; **Supplementary Table 1**). Notably, petunidin-3-(cis/trans-*p*-coumaroyl)-rutinoside-5-glucoside accumulated to significantly higher levels in the peels of IR and YP, compared to RR, PR, and YR (**Figure 8A**). This compound has previously been identified as a major anthocyanin in purple tomato varieties (Mes et al., 2008; Tsugawa et al., 2015; Su et al., 2016; Wang et al., 2017, 2020), suggesting that the anthocyanin biosynthesis pathways in IR and YP are comparable to those in other purple-fruited cultivars. In addition, peonidin and malvidin derivatives, including malvin, were detected exclusively in the peels of IR and YP, but were absent in the other lines (**Figure 8A**). These findings are consistent with the total anthocyanin content, which showed that pigment accumulation primarily occurred in IR and YP peels (**Figure 7A**), and with transcriptomic data showing high expression of genes promoting petunidin biosynthesis (**Figure 5**). Moderate expression of *F3′H* and *AnthOMT*, which are required for the biosynthesis of peonidin and malvin, respectively, was also observed in IR and YP peels, supporting the presence of these compounds (**Figure 5**).

To quantify individual carotenoids, we conducted targeted UPLC-DAD-MS analysis for lycopene, lutein, α-carotene, and β-carotene (**Figure 8B**). Lycopene was abundant in both peel and flesh tissues of IR, RR, and PR, with concentrations ranging from 1.0 to 1.5 µg/g DW at Stage 3 and slightly increased to 1.0–1.7 µg/g DW at Stage 4 (**Figure 8B**). In contrast, lycopene was undetectable in both peel and flesh samples of YR and YP at both developmental stages (**Figure 8B**). This is likely attributable to the *PSY1/Unknown* allele and the reduced expression of the wild-type *PSY1* mRNA transcripts in YR and YP tissues (**Figures 1F**; **6B**), which may limit phytoene production and consequently reduce lycopene accumulation. Similarly, β-carotene levels were elevated in both the peel and flesh of IR, RR, and PR at Stages 3 and 4, ranging from 4 to 8 µg/g DW (**Figure 8B**). In contrast, YR and YP exhibited substantially lower β-carotene concentrations, with 2–3 µg/g DW in the peel and less than 1 µg/g DW in the flesh (**Figure 8B**). This pattern is consistent with the lycopene profile, as lycopene is enzymatically converted to β-carotene in chromoplasts via *CYC-B*. The reduced expression of *CYC-B* in the flesh of YR and YP, relative to their respective peel tissues, may further limit this conversion in the flesh (**Figure 6B**).

Since lycopene can also be converted into δ-carotene and subsequently α-carotene via *LCY-E* and *LCY-B* (**Figure 6A**), we quantified α-carotene levels in all tomato lines. Interestingly, the highest α-carotene content was detected in the peels of YR and YP, reaching approximately 0.4–0.5 µg/g DW (**Figure 8B**). In contrast, IR and PR peels contained lower levels (~0.3 µg/g DW), while α-carotene was undetectable in RR peels (**Figure 8B**). Flesh tissues of IR, RR, and PR lacked detectable α-carotene, whereas YR and YP flesh contained 0.1–0.2 µg/g DW (**Figure 8B**). Overall, α-carotene was present at relatively low levels in those tissues, consistent with previous reports that it is not a major carotenoid in most tomato lines (Ali et al., 2020). This observation is also supported by the generally low expression of *LCY-B* and *LCY-E* across genotypes (**Figure 6B**). The elevated α-carotene content observed in YR and YP may reflect a shift in metabolic flux caused by the *PSY1/Unknown* allele and reduced *CYC-B* expression. Specifically, the reduced expression of the wild-type *PSY1* mRNA transcripts from the *PSY1/Unknown* allele likely impaired lycopene biosynthesis in YR and YP (**Figures 1F**; **6B**). Concurrently, the downregulation of *CYC-B* expression in these lines may have limited the conversion of lycopene to β-carotene, thereby redirecting the metabolic flux toward δ-carotene and subsequently α-carotene biosynthesis (**Figures 6B**, **8B**). Although *LCY-E* expression was comparable across all lines (**Figure 6B**), this redistribution of metabolic flux likely accounts for the increased accumulation of α-carotene in YR and YP. Finally, lutein levels were also examined. Lutein accumulated more abundantly in peels than in flesh across all lines, with peel concentrations ranging from 8 to 20 µg/g DW and flesh concentrations from 2 to 5 µg/g DW (**Figure 8B**). The generally lower expression of *CYP97A3* and *CYP97C1* in flesh tissues likely limited the conversion of α-carotene to lutein in these tissues (**Figure 6B**).

## Discussion

The generation of tomato lines with diverse fruit coloration in the IR genetic background demonstrates the effectiveness of combining genome editing with conventional breeding to manipulate key regulators of carotenoid and flavonoid/anthocyanin biosynthesis. The distinct pigmentation patterns observed in the derived lines, RR, PR, YR, and YP, are directly attributed to targeted modifications in *Aft*, *Myb12*, and *PSY1* (**Figures 1A–F**; **Table 1**). Disruption of *Aft* in RR, PR, and YR markedly reduced anthocyanin accumulation in fruit peel, in agreement with prior studies establishing *Aft* as a master regulator of flavonoid biosynthesis (Mes et al., 2008; Gonzali et al., 2009; Sun et al., 2020; Yan et al., 2020). Additional knockout of *Myb12* in PR suppressed naringenin chalcone production, shifting pigmentation toward pink fruits (Deng et al., 2018; Zhu et al., 2018; Yang et al., 2023), whereas the *PSY1/Unknown* allele in YR and YP impaired carotenoid biosynthesis, producing yellow-fleshed fruits (Yuan et al., 2008; Kang et al., 2014). Collectively, these results illustrate that targeted perturbation of a small number of regulatory genes can effectively reprogram fruit pigmentation without altering overall fruit morphology, thus offering an efficient route to diversify tomato appearance.

Unlike common cultivars such as Ailsa Craig, which do not accumulate anthocyanins in fruit peel (Povero et al., 2011; Sun et al., 2020), RR, PR, and YR retained basal anthocyanin production despite their reduced purple pigmentation at ripeness. Low but detectable levels of anthocyanins were observed in their peels (**Figure 7A**), and these lines displayed dark-green to purple coloration prior to the ‘pink stage’ (**Supplementary Figure 3**). Moreover, they exhibited purple stems and leaves (**Supplementary Figure 4**), consistent with *atv* genotypes (Mes et al., 2008; Colanero et al., 2018). The *Atv* gene encodes an R3-MYB protein that represses anthocyanin biosynthesis (Cao et al., 2017; Colanero et al., 2018; Sun et al., 2020; Yan et al., 2020), whereas the recessive *atv* alleles in RR, PR, and YR alleviate this repression. This explains their limited, early-stage anthocyanin production. However, total anthocyanin content in peel of these lines remained below 10% of that in IR and YP (**Figure 7A**), insufficient to significantly affect peel coloration at the fully ripened stage (**Figure 1A**; **Supplementary Figure 3**). These observations highlight the epistatic interactions between *Aft* and *atv*, as well as the tissue- and stage-specific regulation of anthocyanin biosynthesis in tomato.

Transcriptomic analyses provided deeper insight into how these genetic modifications shaped fruit development. Peel and flesh tissues followed distinct developmental trajectories, with divergence becoming more pronounced at Stage 3, corresponding to the onset of ripening (**Supplementary Figures 3**, **6**). Most DEGs were line-specific, reflecting the strong influence of the introduced alleles on global transcriptional profiles (**Figure 2**). Notably, YP exhibited the largest number of DEGs at Stage 2, suggesting that the *PSY1/Unknown* allele exerts its strongest regulatory effect during the early phase of carotenoid accumulation (**Figures 3**, **4**). Interestingly, the majority of these transcriptional changes involved fundamental physiological processes such as carbohydrate and acid metabolism, cell cycle, as well as oxidative stress, rather than direct pigment biosynthesis (**Figures 3**, **4**). Furthermore, the distinct transcriptomic difference observed in YP was absent in YR, despite their genetic similarity except for the *aft* mutant allele (**Table 1**). This suggests a potential antagonistic relationship between *Aft* and *PSY1* in regulating these physiological processes, in addition to their known functions in anthocyanin and carotenoid biosynthesis, respectively.

As *AFT* and *Myb12* were previously found to be transcription factors (Ballester et al., 2010; Yan et al., 2020), our study has further showed that mutations to either gene (*aft* in RR, as compared to IR; *myb12* in PR, as compared to RR) have contributed to changes in the transcriptome (**Figures 2**, **4**) and metabolome (**Figures 1D**, **5**, **8**). The effects of both mutations were clear in the expression of flavonoid biosynthesis genes. Mutation of *aft* in RR peel resulted in the suppression of *PAL6*, *F3’H-3*, *F3’5’H*, *DFR-1*, *ANS*, and *AnthOMT*, as compared to IR (**Figure 5B**). The additional mutation of *myb12* in PR peel caused further inhibition of *4CL-2*, *CHS1/2*, *F3H*, *F3’H-2*, and *RT*, in comparison to RR (**Figure 5B**). Interestingly, the double mutations of *aft* and *myb12* in PR partially rescued the suppressed expression of some genes, including *PAL2* and *4CL-5* in stage 1 PR peel (**Figure 5B**). This implies that genome editing of multiple transcription factors may not result in a strictly additive effect on the transcriptome. This is further supported by the GO terms identified from RR and PR (**Figures 3**, **4**). While several GO terms were shared by both genotypes (e.g. “protein complex oligomerization”, “response to hydrogen peroxide”, and “response to osmotic stress” in RR and PR flesh samples), many of the identified GO terms were unique to either RR or PR, thereby indicating transcriptomic divergence between genotypes.

GO enrichment analyses further revealed functional divergence among the tomato lines (**Figures 3**, **4**). Notably, the flavonoid and anthocyanin biosynthetic pathways were up-regulated in IR and YP, whereas they were down-regulated in RR, PR, and YR (**Figures 3**, **4**). This expression pattern is consistent with the observed differences in peel coloration (**Figure 1A**; **Supplementary Figure 3**) and the elevated anthocyanin accumulation detected in IR and YP (**Figures 7A**, **8A**). Although DEGs associated with flavonoid and anthocyanin biosynthesis were also identified in flesh tissues (**Figure 4**), anthocyanin levels remained comparably low across all lines (**Figure 7A**). Previous studies have shown that anthocyanin accumulation in the fruit peel of IR is light-dependent and requires the activity of the bZIP transcription factor SlHY5 (Sun et al., 2020).

Further, integration of transcriptomic and metabolomic datasets confirmed that gene expression changes directly influenced pigment accumulation. High expression of key flavonoid and anthocyanin biosynthetic genes, including *PAL3*, *CHS1/2*, *F3′5′H*, *DFR-1*, and *ANS*, in IR and YP peel tissues aligned with their high anthocyanin contents, which was dominated by delphinidins, petunidins, and malvidins (**Figures 5**, **8A**). In contrast, reduced expression of these genes in RR, PR, and YR corresponded with weak or absent purple pigmentation (**Figures 5**, **8A**). Carotenoid profiles were shaped primarily by *PSY1* functionality. IR, RR, and PR accumulated high levels of lycopene and β-carotene in both peel and flesh, whereas YR and YP (containing the *PSY1/Unknown* mutant allele) accumulated much lower levels, consistent with their yellow flesh phenotype (**Figure 8B**). Importantly, altered expression of downstream genes such as *CYC-B* and cytochrome P450s further differentiated carotenoid composition across tissues, underscoring the complexity of the regulatory network controlling pigment biosynthesis (**Figures 6**, **8B**).

An intriguing observation was the elevated α-carotene content in YR and YP (**Figure 8B**). This accumulation appears to result from metabolic flux redistribution following impaired *PSY1* function and reduced *CYC-B* expression (**Figure 6B**). With lycopene synthesis constrained and conversion to β-carotene limited, precursors may be redirected toward δ-carotene and subsequently α-carotene biosynthesis. This metabolic flexibility illustrates the robustness of the carotenoid pathway under genetic perturbation. From a nutritional perspective, the increase in α-carotene is noteworthy because α-carotene, like β-carotene, is a provitamin A carotenoid with recognized health-promoting effects (Blaner, 2020). Thus, these findings reveal that the *PSY1/Unknown* allele in YR and YP can not only diversify tomato color phenotypes but also fine-tune the nutritional composition of fruits. Such outcomes may be particularly valuable in efforts to develop biofortified crops with improved dietary contributions.

The broader implications of these results extend to both breeding strategies and horticultural production systems. From a breeding perspective, the study illustrates how rational design, guided by prior knowledge of transcriptional regulators and key enzymes, can generate predictable outcomes in fruit pigmentation. The ability to produce tomatoes with customized combinations of peel and flesh pigmentation, while maintaining desirable horticultural traits, provides a versatile platform for diversifying tomato germplasm. In addition, these lines provide valuable materials for dissecting gene regulatory networks underlying pigment biosynthesis, as they combine multiple allelic variants in defined genetic backgrounds. In the context of indoor farming and controlled environment agriculture, the newly developed lines offer additional advantages. Sharing similar genetic backgrounds, growth habits, flowering times, and developmental durations, these lines can be cultivated synchronously under identical indoor conditions, enhancing production efficiency and reducing facility management costs.

In summary, this study demonstrates that targeted manipulation of *Aft*, *Myb12*, and *PSY1* in the Indigo Rose background enables precise modulation of fruit peel and flesh pigmentation, flavonoid and carotenoid biosynthesis, and associated metabolic profiles. The resulting lines, RR, PR, YR, and YP, exhibit distinct and predictable color phenotypes, underpinned by tissue- and stage-specific transcriptional changes. Integration of transcriptomic and metabolomic analyses confirmed that these genetic modifications directly shape pigment accumulation and metabolic fluxes, revealing new insights into the regulatory networks controlling tomato fruit coloration. Collectively, these findings establish a robust framework for genome editing and breeding strategies aimed at producing visually appealing, nutritionally enriched, and specialty tomato varieties suitable for both conventional and indoor cultivation systems.

## Data Availability

The original RNA-Seq data generated in this study have been deposited in the National Center for Biotechnology Information (NCBI) Sequence Read Archive (SRA) and are publicly available under accession number PRJNA1484193.
