# Supplementary material for: Developing new tomato lines in Indigo Rose genetic background with differential anthocyanin and carotenoid production in fruits
Source: Front Plant Sci. 2026 Jul 13;17:1877977. doi: 10.3389/fpls.2026.1877977 (PMC13403201; doi:10.3389/fpls.2026.1877977)
Supplement: Supplementary file 1 [file SupplementaryFile1.docx]

**Developing new tomato lines in Indigo Rose genetic background with differential anthocyanin and carotenoid production in fruits**

Dongsheng Tian^1,#^, Benny Jian Rong Sng^1,#^, Ignatius Ren Kai Phang^1,#^, Sing Hui Leong^1,#^, Raji Mohan^1,#^, Shilu Zhang^1,#^, Yuejing Gui^1^, In-Cheol Jang^1,2,*^ and Zhongchao Yin^1,2,*^

^1^Temasek Life Sciences Laboratory, 1 Research Link, National University of Singapore, Singapore 117604, Republic of Singapore.

^2^Department of Biological Sciences, 14 Science Drive, National University of Singapore, Singapore 117543, Republic of Singapore.

**Supplementary figures**


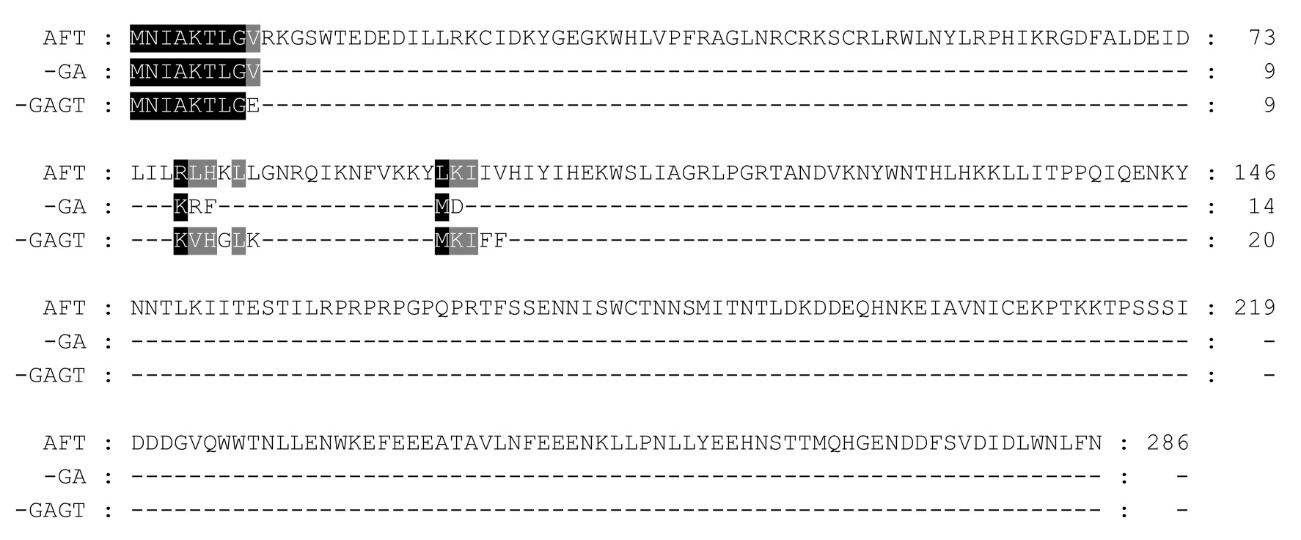


**Figure S1. Amino acid sequence alignment of AFT and its mutant variants.**
Amino acid sequences of AFT and its CRISPR/Cas9-induced mutant variants were aligned using the MAFFT algorithm implemented in MegAlign Pro and visualized with GeneDoc. Residues that are fully conserved across all aligned sequences are highlighted in black, while those conserved in most sequences are shaded in grey. The -GA and -GAGT variants represent two AFT mutant alleles generated through CRISPR/Cas9-mediated mutagenesis.


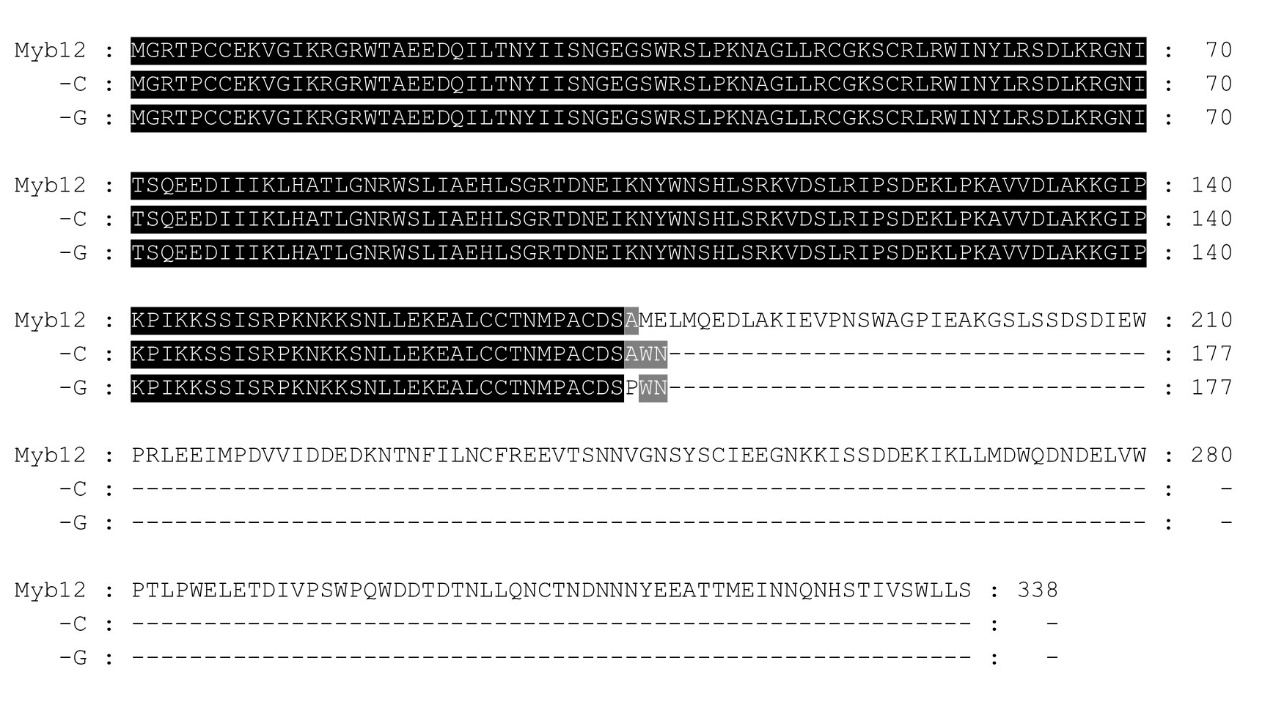


**Figure S2. Amino acid sequence alignment of Myb12 and its mutant variants.**
Amino acid sequences of Myb12 and its CRISPR/Cas9-induced mutant variants were aligned using the MAFFT algorithm implemented in MegAlign Pro and visualized with GeneDoc. Residues that are fully conserved across all aligned sequences are highlighted in black, while those conserved in most sequences are shaded in grey. The -C and -G variants represent two Myb12 mutant alleles generated through CRISPR/Cas9-mediated mutagenesis.

**
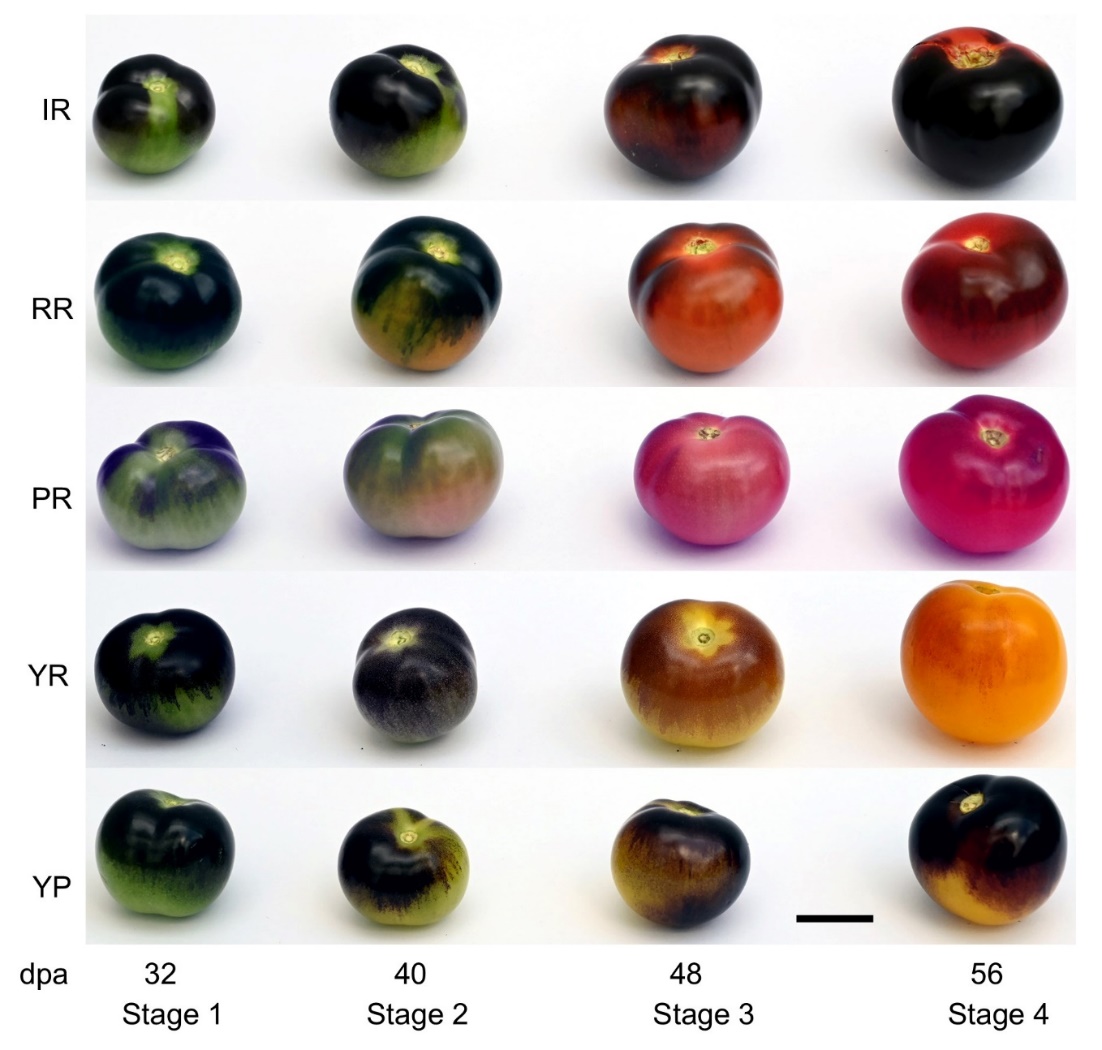
**

**Figure S3.**  **Fruit samples used for RNA-seq and metabolic analyses**

Samples collected at 32, 40, and 48 dpa were used for transcriptome analysis, while those collected at 48 and 56 dpa were used for metabolic profiling. The corresponding developmental stages of fruits are indicated below each dpa. Scale bar = 2 cm.


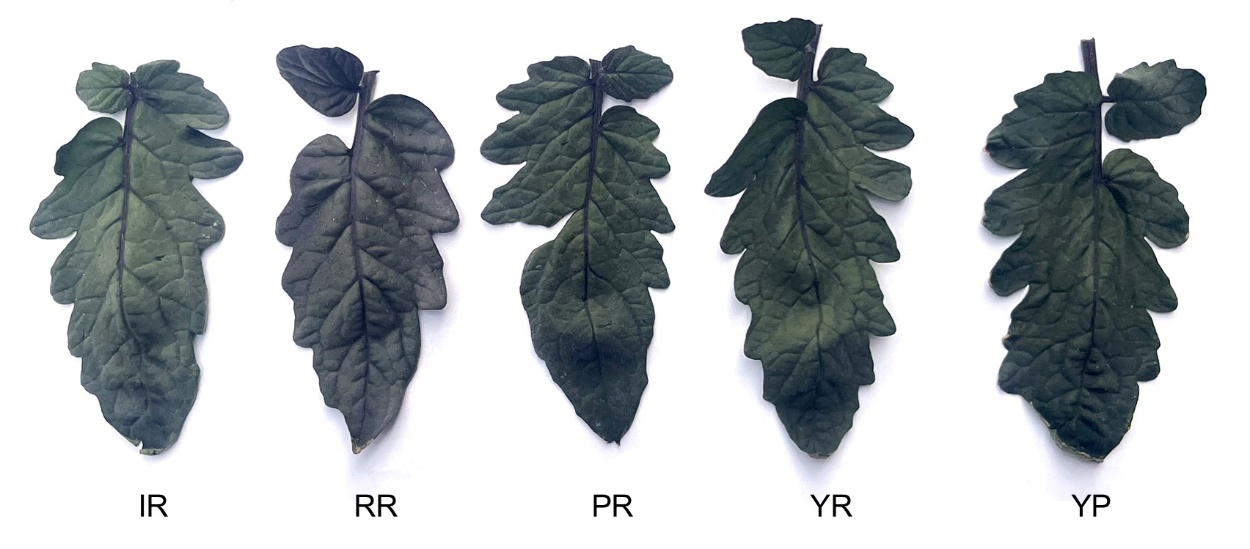


**Figure S4. Leaf phenotypes of tomato lines.**

Abbreviations: IR, Indigo Rose; RR, Red Rose; PR, Pink Rose; YR, Yellow Rose; YP, Yellow-Purple Rose.


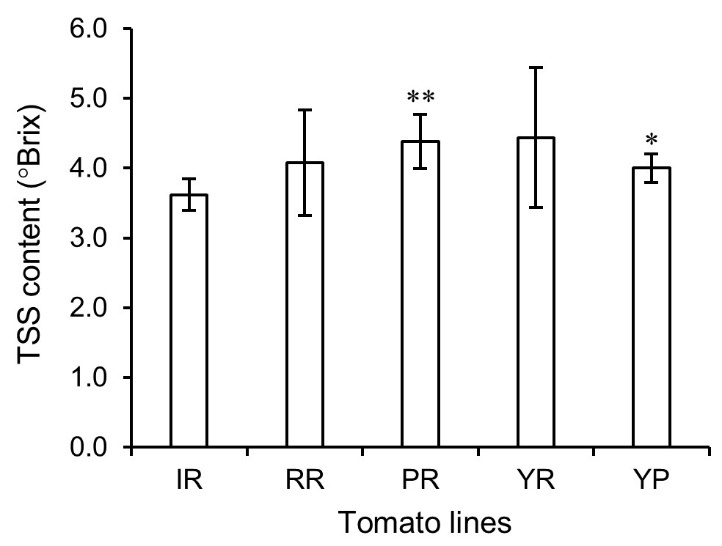


**Figure S5. Total soluble solids (TSS) content in tomato fruits.**
TSS content in tomato fruits at 56 dpa was measured using a handheld refractometer (model R9500, REED Instruments). Results are expressed in Brix degrees. Asterisks indicate statistically significant differences between IR and individual variant (**, p ≤ 0.01; *, 0.01 < p ≤ 0.05; Welch’s *t*-test). Abbreviations: IR, Indigo Rose; RR, Red Rose; PR, Pink Rose; YR, Yellow Rose; YP, Yellow-Purple Rose.


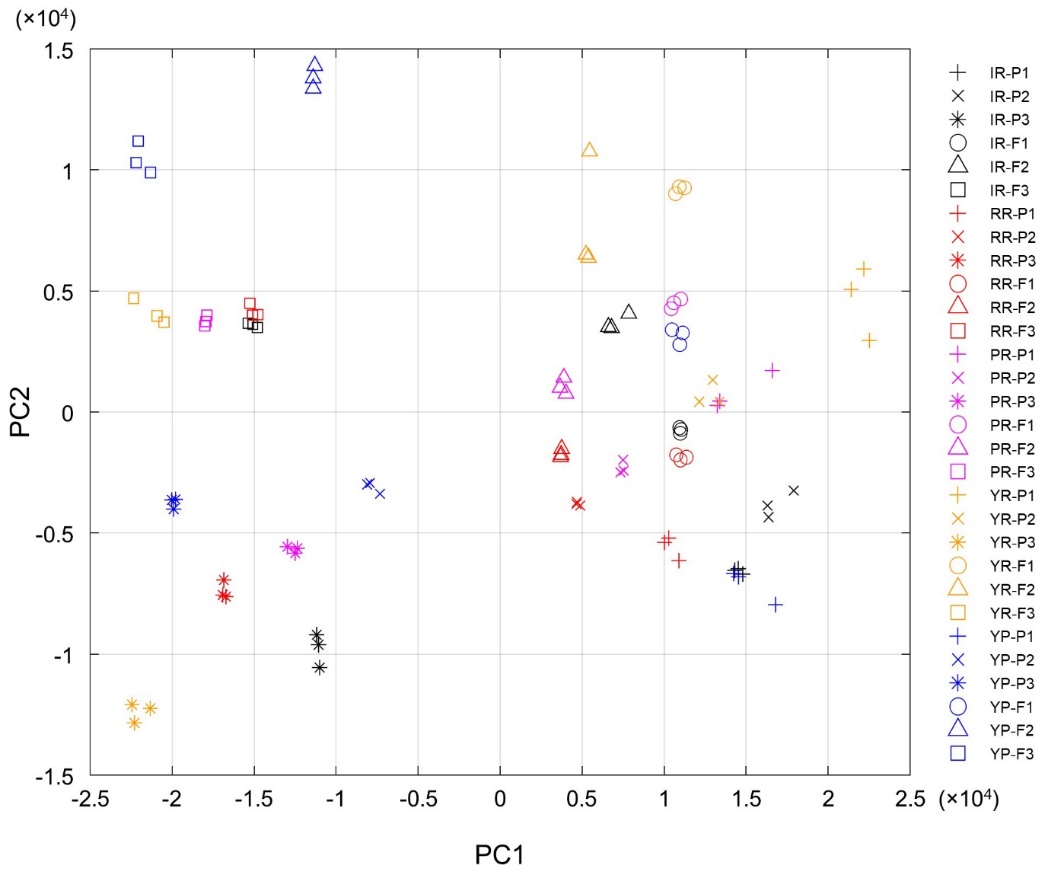


**Figure S6. Principal component analysis (PCA) of RNA-seq profiles from all tomato samples.**
The PCA plot illustrates the transcriptomic variation among samples based on RNA-seq analysis. Sample types are indicated as peel (P) or flesh (F), followed by the developmental stage of the tomato fruit (e.g., P1, peel at Stage 1*).* Abbreviations: IR, Indigo Rose*;* RR*,* Red Rose*;* PR*,* Pink Rose*;* YR*,* Yellow Rose*;* YP*,* Yellow-Purple Rose*.*


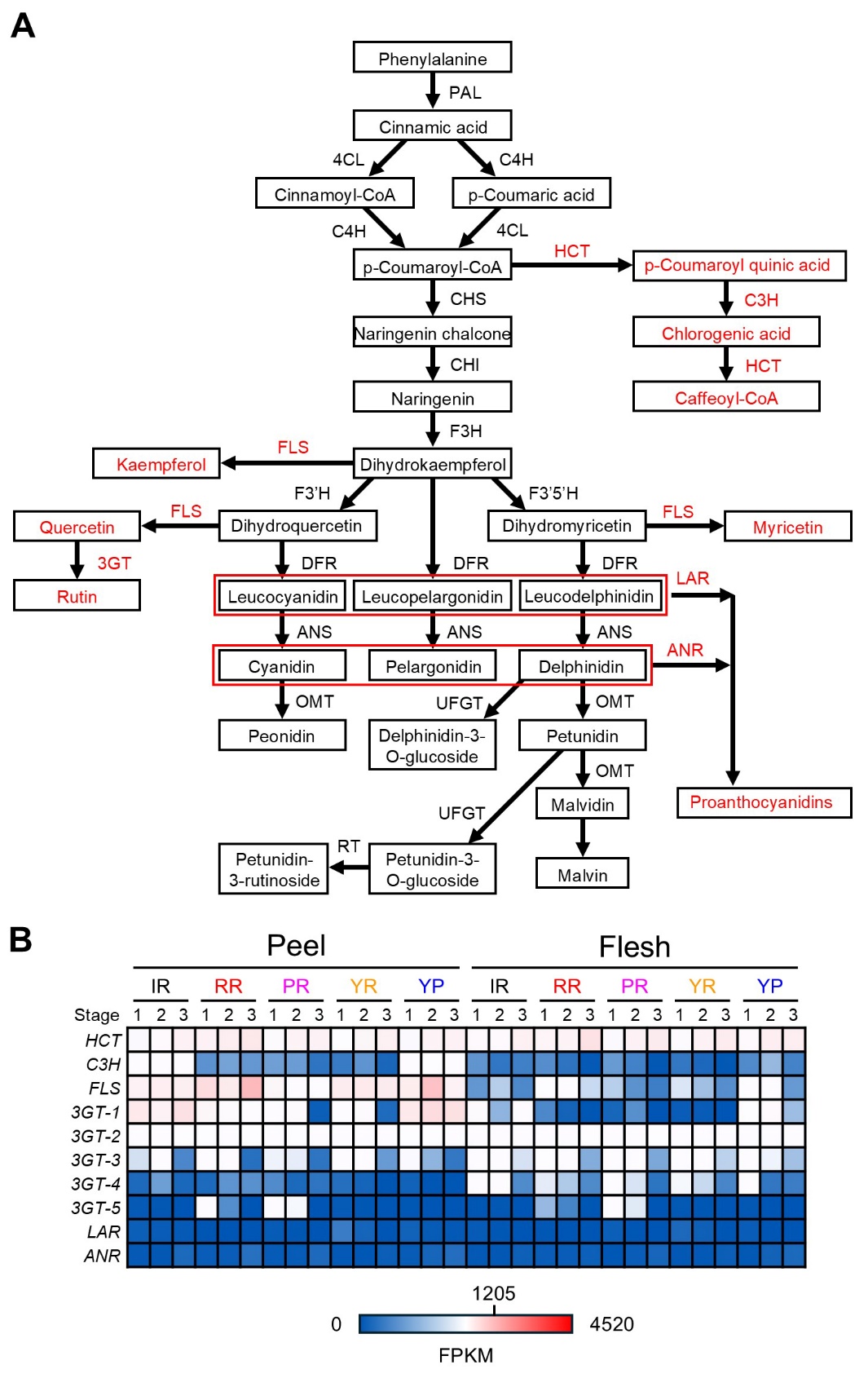


**Figure S7. Expression of genes involved in flavonoid and flavonoid-derived compound biosynthesis in tomato fruits.**
(**A**) Schematic representation of the flavonoid and flavonoid-derived compound biosynthesis pathways in tomato. Enzymes and compounds associated with flavonoid derivatives are indicated in red. Abbreviations: ANR, anthocyanidin reductase; ANS, anthocyanin synthase; AnthOMT, anthocyanin-targeting O-methyltransferase; C3H, coumaroyl CoA 3-hydroxylase; C4H, cinnamate 4-hydroxylase; CHI, chalcone isomerase; CHS, chalcone synthase; DFR, dihydroflavonol 4-reductase; FLS, flavonol synthase; F3H, flavanone 3-hydroxylase; F3’5’H, flavonoid 3',5'-hydroxylase; F3'H, flavonoid 3'-hydroxylase; HCT, hydroxycinnamoyl CoA shikimate/quinate hydroxycinnamoyl transferase; LAR, leucoanthocyanidin reductase; PAL, Phenylalanine ammonia-lyase; RT, rhamnosyltransferase; UFGT, UDP-glucose:flavonoid-3-O-glucosyltransferase; 4CL, 4-coumarate:CoA ligase; 3GT, quercetin-3-O-glucosyl transferase. (**B**) Heatmap showing the expression levels of the genes highlighted in (**A**). Expression values are presented using the same color scale as in Figure 7B. The actual FPKM values range from 0 to 1205. Abbreviations: FPKM, fragments per kilobase of transcript per million mapped reads; IR, Indigo Rose; RR, Red Rose; PR, Pink Rose; YR, Yellow Rose; YP, Yellow-Purple Rose.


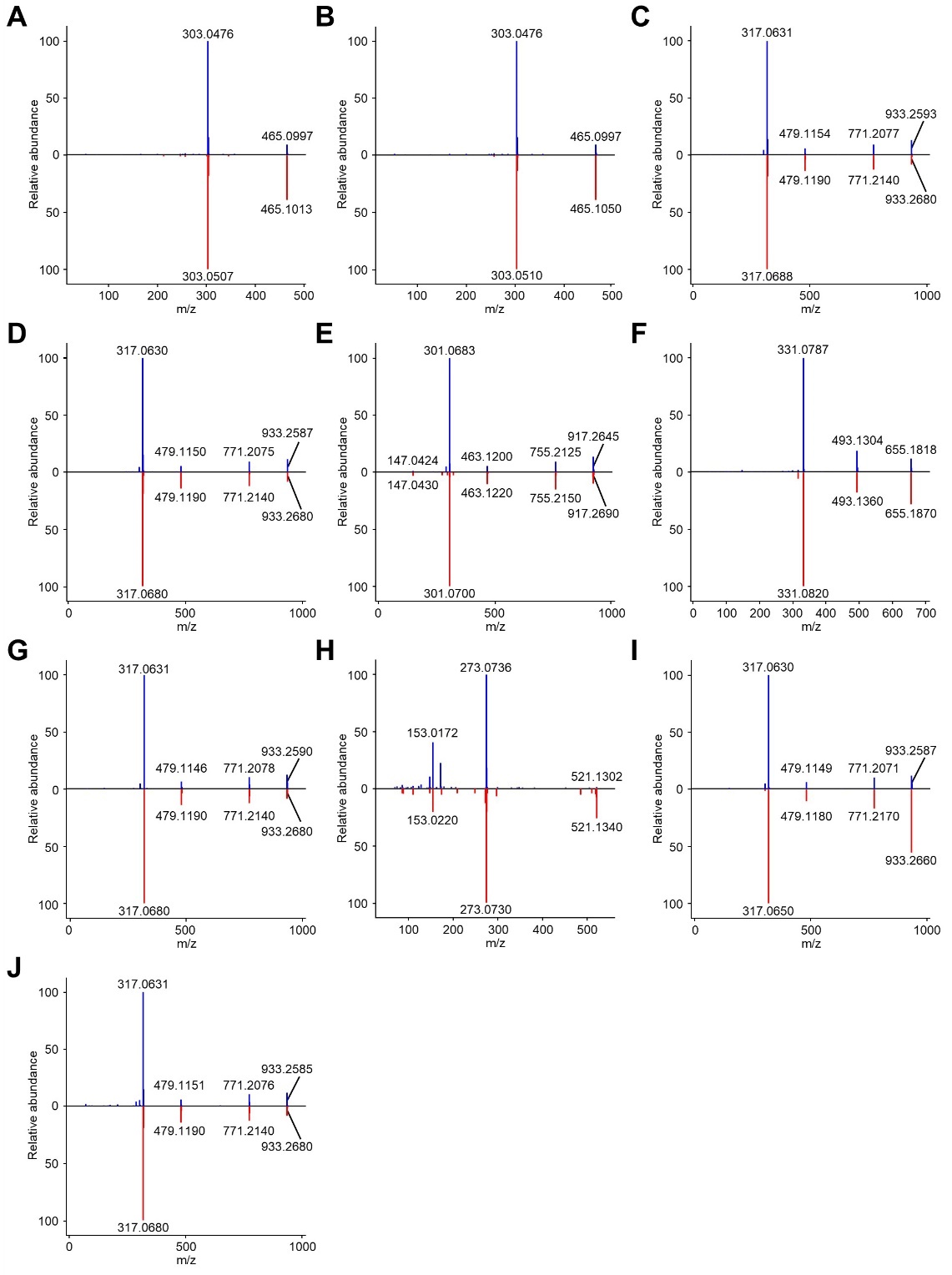


**Figure S8. Identification of individual anthocyanins using UHPLC-Orbitrap MS/MS analysis.**
Representative MS/MS spectra of anthocyanins detected in the samples (top, blue) are shown alongside their corresponding reference spectra retrieved from spectral databases (bottom, red). The identified anthocyanins include: (**A**) Delphinidin 3-galactoside, (**B**) Delphinidin 3-glucoside, (**C**) Petunidin-3-(cis-p-coumaroyl)-rutinoside-5-glucoside, (**D**) Petunidin-3-(trans-p-coumaroyl)-rutinoside-5-glucoside, (**E**) Peonidin 3-O-[6-O-(4-O-(E)-p-coumaroyl-O-α-rhamnopyranosyl)-β-glucopyranoside]-5-O-β-glucopyranoside, (**F**) Malvin, (**G**) Petanin (isomer 1), (**H**) Anthocyanidin base + 3O, O-malonylHex, (**I**) Petunidin-3-O-(6''-O-(4'''-O*-*E*-*p-coumaroyl)-α-rhamnopyranosyl-β-glucopyranosyl)-5-O-β-glucopyranoside, and (**J**) Petanin (isomer 2).
